# Selective Carbon Material Engineering for Improved MEMS and NEMS

**DOI:** 10.3390/mi10080539

**Published:** 2019-08-16

**Authors:** Stephane Neuville

**Affiliations:** Independent Consultant, F-77165 Cuisy, France; Steph.neuville709@orange.fr

**Keywords:** carbon-based material, carbon structure differentiation, NEMS quality, higher performances, revised Raman characterization, quantum electronic activation, carbon phase transition

## Abstract

The development of micro and nano electromechanical systems and achievement of higher performances with increased quality and life time is confronted to searching and mastering of material with superior properties and quality. Those can affect many aspects of the MEMS, NEMS and MOMS design including geometric tolerances and reproducibility of many specific solid-state structures and properties. Among those: Mechanical, adhesion, thermal and chemical stability, electrical and heat conductance, optical, optoelectronic and semiconducting properties, porosity, bulk and surface properties. They can be affected by different kinds of phase transformations and degrading, which greatly depends on the conditions of use and the way the materials have been selected, elaborated, modified and assembled. Distribution of these properties cover several orders of magnitude and depend on the design, actually achieved structure, type and number of defects. It is then essential to be well aware about all these, and to distinguish and characterize all features that are able to affect the results. For this achievement, we point out and discuss the necessity to take into account several recently revisited fundamentals on carbon atomic rearrangement and revised carbon Raman spectroscopy characterizing in addition to several other aspects we will briefly describe. Correctly selected and implemented, these carbon materials can then open new routes for many new and more performing microsystems including improved energy generation, storage and conversion, 2D superconductivity, light switches, light pipes and quantum devices and with new improved sensor and mechanical functions and biomedical applications.

## 1. Introduction

Although, micro, nano electromechanical and optomechanical systems are still often confronted to the lack of quality and longer life time and to the search of extended higher performances [1], huge progress has been recently achieved in MEMS and NEMS technology in using more performing carbon-based materials, which are presenting a large panel of various superior properties concerning their mechanical properties, such as young modulus, Poisson’s ratio, fracture strength of nanocrystalline diamond for instance [2], tribological, electric, semicon, piezoelectric, heat conducting and optical/optoelectrical properties [3,4], diamond micro and nano resonators [5,6], piezo-resistivity obtained with carbon nanotubes [7], diamond-like carbon MEMS sensors [8] and many more which are making use of functionalized graphenic and related materials [9,10,11,12,13,14,15,16,17,18,19,20].

Key hurdles currently preventing the commercial application of many NEMS devices include low-yields and high device quality variability, concerning structure, physical and chemical stability, nucleation, adhesion, different kinds of internal and interface stress, tribology and wear rates, contamination diffusion barrier properties, stability and reproducibility of surface functionalization [16,17,18,19]. Before NEMS devices can actually be industrially implemented, reasonable integrations of carbon-based products must be created [21]. Next, the challenge to overcome is understanding carbon-based materials properties, with which efficient and durable NEMS with low failure rates can be achieved [22,23].

Basically, those can often be improved in using selectively different kinds of carbon materials including the different sorts of diamond, tetrahedral carbon, DLC, GLC, glassy carbon, nanowires and nanotubes and graphenic materials of specific properties [9] and we discuss that in more details in this study. Those have intrinsic properties, which are distributed over several order of magnitudes and have to be well distinguished from each other [2,3,12,13,21].

Limiting factors of their implementation are generally a consequence of degraded structure and defects and possible induced phase transformation which are depending on their elaborating device and application environment [23,24]. It then appears necessary to also have all sorts of appropriate characterizing devices, which have to be used for the different process optimization steps. Further on it is then also to be considered well understood the aspects of surface preparation and processing, nucleation and growth mechanisms and especially the phase transformation they can be subject and which have been generally neglected and omitted to be considered although appearing of greatest importance [12,13,25,26,27].

Necessary mastering of differentiate carbon material depositing and characterizing can be achieved with recently revisited fundamentals on carbon atomic rearrangement [25] and carbon Raman spectroscopy [26]. In addition to several other revisited subjects, we briefly recall concerning diamond-like carbon coatings [27], energy storage and conversion using different kinds of carbon-based materials [28], superconductivity (because of some analogy with electron ballistic properties in graphenic materials [29], for which carbon materials need to be correctly selected and associated. This appears all the more important to be achieved, considering those, can open new routes for improved microsystems and 2D devices concerning mechanical functions, light switches, light pipes and quantum calculation devices [30].

## 2. Brief Review on Main MEMS and NEMS Characteristics

### 2.1. Early Stage of Microelectromechanical Systems (MEMS)

MEMS is the technology of microscopic devices, particularly those with moving parts smaller than a human hair with outstanding accelerating practical application interest during the last decades and which have rapidly achieved actuator dimensions in the 100 nm range, before becoming much smaller with nano-technologic means. They are now used for many applications, and a future form of NEMS is expected to exceed the IC industry in both size and impact on society [1].

These devices replace bulky actuators and sensors with a micron scale equivalent that can be produced in large quantities by the fabrication process used in integrated circuits. They reduce cost, bulk, weight and power consumption while increasing performance, production volume and functionality by orders of magnitude [21,23].

Those are concerning multidisciplinary fields in the areas of engineering, chemistry, material science, physics, and any specialized field for applications in bioengineering or medicine. Their future holds revolutionary breakthroughs in a wide range of: Nanocircuits, actuators (piezo-electrostatic, shape memory, electromagnetic), sensors, radar, locators, materials, imaging, nanocontact and nano-relays [31,32,33,34,35,36,37,38,39,40,41], micromotors and micropumps [42,43,44,45,46,47,48,49], optical functions and optoelectronic devices, such as switches, integrated energy harvesting, wave length filtering, optical grating switch and optical sensors—these MEMS are called micro optical electromechanical systems (MOMS) [50,51,52], energy storage [28,53], nanocapacitors, data storage and nano-computers [1,30,54,55,56] including 2D superconducting devices to be used for quantum computers [29], and such as, bio-functionalizing, etc. [1,11,16,57,58].

### 2.2. MEMS Fab

MEMS became practical once they could be fabricated using modified semiconductor device fabrication technologies, normally used to make electronics. They usually consist of a central unit that processes data (the microprocessor) and several components that interact with the surroundings such as microsensors [1,21,30]. The fabrication of MEMS evolved from the process technology in semiconductor device fabrication, i.e., the basic techniques are the deposition of material layers, patterning by photolithography and etching to produce the required shapes and to which different types of bulk and surface micromachining of different materials can be associated [59,60,61].

The addition of specific material properties with the availability of inexpensive high-quality materials, and ability to incorporate electronic functionality make silicon attractive for a wide variety of MEMS applications. However, considering the large surface area to volume ratio of MEMS, and complex interface mechanical, chemical, electrical and electro-magnetic phenomena, their design requests particular attention and several other materials have been also considered for MEMS manufacturing, such as polymers, metals, and ceramics before considering advanced carbon materials [1,4,5,6,7,8,9,10,11,12,13,14,15,16] for many specific reasons on which we focus on in Section 3, Section 4 and Section 5.

### 2.3. NEMS

#### 2.3.1. Definition and General Features

MEMS technology evolves into smaller nanoelectromechanical systems (NEMS) and nanotechnology [59,60,61,62]. These devices replace bulky actuators and sensors with a micron scale equivalent that can be produced in large quantities by the fabrication process used in integrated circuits in photolithography. They reduce cost, bulk, weight and power consumption while increasing efficiency, performance, production volume and functionality by orders of magnitude. They achieve a reduced size down to 10 nm range and less considering the size of some of their subsystems [63,64]. Miniaturization of MEMS fab could be achieved with two complementary approaches. (A) Top-down approach uses the traditional microfabrication methods. While being limited by the resolution of these methods, it allows a large degree of control over the resulting structures such as nanowires, nanorods, and patterned nanostructures are fabricated from metallic thin films or etched semiconductor layers [62]. (B) Bottom-up approaches, in contrast, use the chemical properties of single molecules to cause single-molecule components to self-organize or self-assemble into some useful conformation, or rely on positional assembly and allows fabrication of much smaller structures [63]. Those can be made at the VLSI scale, possibly co-integrated with CMOS well suited for autonomous, highly sensitive or dense sensors. They include complex gas portable recognition systems, mass spectrometry, or bio-sensors [11,12,13,14] and open several opportunities for integrated solutions in emerging domains as chemical analysis and life science [16,21,22,23,24,30,57,58].

#### 2.3.2. Early NEMS Application Fundamentals

Nanoscale mechanical sensors offer a greatly enhanced performance that is unattainable with microscale devices [59,60,61,62,63,64,65,66,67]: Ultrasensitive sensors [68], high quality factor diamond resonators [69], high mass and spatial resolution basing on mechanical resonance and cantilever vibration up to high frequencies [70], which is enabling chemisorption measurements in air at room temperature, with very high mass resolution below 1 atto-gram (10–18 g) [71]. Further on, high-sensitive liquid/airflow meter could be produced [72], nano ph sensor [73] and nano-molecular machines [74,75,76,77,78,79,80].

A molecular machine is a group of molecular components that are able to produce quasi-mechanical movements when exposed to specific stimuli.

There are three categories of the molecular machines, namely natural or biological, synthetic, and natural-synthetic hybrid machines [81,82,83]. Synthetic molecular machine includes motors, propellers, switches, shuttles, tweezers, sensors and logic gates. Biological motors convert chemical energy into linear or rotary motion as well as controlling many biological functions. Examples of linear motions: Proteins, muscle contraction, intracellular transport, signal transduction, ATP synthesis, membrane translocation proteins and the flagella motor. Natural-synthetic hybrid systems are mechanical motors inspired from DNA duplication and partition [83,84,85,86,87,88].

The NEMS technology is distinguished from molecular nanotechnology or molecular electronics in that the latter must also consider surface chemistry and solid-state phonon and electronic quantum mechanical aspects which can affect mechanical, electric and optoelectronic properties, friction and that can cause high signal/noise ratio ([89,90,91,92]. Mechanical deformation and electrical contact properties and adhesion between carbon nanotubes are important aspects of their quality and dynamic performances [93,94,95,96,97,98] and explaining why they must be selected and controlled upon their characteristics, size and defect content.

In spite of already numerous probated applications many potential others corresponding to bench lab prototypes could not yet be sufficiently mastered on their quality, reliability and life time in consequence of insufficiently understood fundamentals [21,22,23]. Among them, those concerning stability and material structure modification being induced by local quantum electronic effects which used to be ignored up to recent past [25,99] and another important quantum mechanical revisited aspect concerning the characterization of carbon material: The recently revised Raman fundamentals with which carbon material structure and their defects can be better and more correctly sorted out [26] and we recall and discuss this in more details next in Section 3, Section 4 and Section 5.

Considering selective electronic activation of semiconducting surface material caused by adsorption and chemisorption and with which transversal polarization effects can appear [100], much sensitive and selective physicochemical interactions can be considered between nanoparticles and the biologic material. This is illustrated with size dependence of Au and Ag particle on different biological metabolism [101,102]. Different corresponding nano-effects are considered for nanomedicine and dentistry applications [103,104] and are also a subject of toxicologic investigations [105,106,107].

## 3. Increased NEMS Performances with Advanced Carbon Material

### 3.1. Progress in NEMS Technologies

They are less mature than that of MEMS due to the difficulty to reliably couple the micro-actuators to the macroscopic world and to achieve requested quality and performances, specially concerning longer life time, higher strength, better adhesion and tribology and better mechanical and chemical stability and better reproducible size, bulk and surface optoelectronic effects and heat/electric conducting properties [21,22,23]. However, owing to the use of different carbon-based materials with corresponding superior solid-state bulk and surface properties and which have been synthetized with more or less empirical means [3,10], the fast-growing number of MEMS/NEMS applications could be achieved. For instance, the scanning tunneling microscopes (STMs), inertial, pressure, thermal, optical, flow, capacitive position sensors, biochips for detection of hazardous chemical and biological agents, high-throughput drug screening and selection, optical switches, valves, RF switches, micro-relays, electronic noses, etc. [1,44,45,61,62,63,64,65,74,108,109,110].

### 3.2. Diamond and Related Materials

#### 3.2.1. Different Categories of Diamond and Diamond-Like Materials

Diamond materials offer great potential for electronic and biomedical application. With very high stiffness, high thermal conductivity, optical transparency range, chemical stability and wear resistance for the diamond-based materials extend their applicability for MEMS/NEMS [24]. Besides, these diamond materials which are nevertheless presenting a large panel of different structures and properties, and which have to be distinguished from each other’s [3,4,5,6,7,8,9,10,11,12,13,14,15,16,17,18,19,20,21,22,23,24,25,26,27,28,29,30,31,32,33,34,35,36,37,38,39,40,41,42,43,44,45,46,47,48,49,50,51,52,53,54,55,56,57,58,59,60,61,62,63,64,65,66,67,68], many other different kinds of diamond-like materials have either to be considered and distinguished from each other’s in so far they often present an underestimated wide range of specific combined properties. Those are depending on specific composite structure, defect, contamination and atomic disorder including physical and chemical, optical and optoelectronic properties, thermal, mechanical, chemical, tribological, and wear resistance, internal mechanical stress, electric properties and their possibility to be doped. Depending also, on their surface micro and nano rugosity, porosity and surface chemisorbing and adsorbing properties, and the way they can be produced [27,111,112,113]. Different categories of diamond and diamond-like carbon (DLC) materials have to be considered:

(a) *Polycrystalline diamond of different crystallite size*, including the hexagonal and epitaxial diamond. To be observed that the denser and smoother micro- and nano-crystalline diamond is almost containing a significant part of graphitic material where more or less ordered/disordered diamond crystallites are imbedded. However, besides interesting tribological properties, those have generally reduced others (optical, optoelectronic, chemical and mechanical) [114,115,116,117,118,119].
(b) *Amorphous diamond and degraded tetrahedral amorphous carbon*, ranging from materials with high internal stress such as the amorphous diamond and ta-C:H and others for which the internal stress could be reduced without graphitic degradation [120,121,122,123,124,125,126].
(c) *Polymeric carbon* [127,128], although not being really diamond-like, but which contain many sp3 carbon chains and have a similar optoelectronic gap.
(d) *Diamond-like a-C:H and composite material* such as diamond-like glassy-carbon CNx and sp3 rich carbon nanowire and fiber [24,27,129,130,131,132,133].

#### 3.2.2. Upholding of Combined Properties

It must be emphasized that all these diamond and diamond-like materials have much different combined properties, concerning their Csp2 and Csp3 content, their thermal and chemical stability, and their simultaneous mechanical, tribological and optoelectronic properties. This is especially concerning the mechanical elastic/plastic/hardness properties which are obtained after an annealing process reducing the internal stress [3]. The resulting material will generally no longer combine higher diffusion barrier, optical and electric and/or dielectric properties. The harder homogeneous isotropic amorphous diamond combines many different superior interesting properties, except its electric conductivity and its internal stress which can only be annealed without degrading the initial properties with particular appropriate means (e.g., hard UV laser annealing) and when the temperature stays lower than the thermal graphitization temperature threshold [120,121,122,123,124].

When it is degraded to a more graphitic material, the amorphous diamond and harder ta-C will generally lose at least at nanoscale their amorphous homogeneity with the formed sp2 clusters (islands of sp2 material within the amorphous sp3 material). Then, they merely correspond to some nanocomposite material containing different adjacent phases and this is often leading to dramatic confusions between materials supposed to belong to the same category, whether being stress annealed or not and presenting important material structures and property differences [27,111,112,113]. Diamond-like carbon produced with non-optimized depositing equipment design and lower optimized depositing process or which is resulting from the thermal annealing of crystalline diamond and from highly stresses harder ta-C has been often considered as an amorphous diamond, although being merely less hard, less homogeneous less smooth, less dense composite material with reduced optical properties and reduced thermal stability (in comparison to the diamond and harder tetrahedral amorphous carbon) [114,115,116,117,118,119,120,121,122,123,124,125].

To be observed that the temperature induced exodiffusion of hydrogen and other gaseous species can induce tensile stress and cracks [27]. This effect can be amplified when a graphitic material is transformed into a denser diamond-like material. These sorts of materials can be obtained with other means than combined pressure/heat and thermal spikes [111,112,113,114,115,116,117,118,119,120,121,122,123,124]. In such a case, contradicting effects have to be considered (either favorizing graphitization, or favorizing diamond-like structures) and which can be in competition to each other [25]. Those are suggested as explaining, for instance, why glassy carbon is not always showing graphenic properties, but can be harder and more diamond-like [132,133]. Similar effects have also to be considered for the growth of carbon nano-fibers of irregular cylindrical structure [134,135,136] and which in contrast to CNT are filled tubes containing also significant amounts of Csp3. For their accurate characterization, anticipating on the next chapter IV, revision of different characterizing such as Raman spectroscopy and interpretation appeared to be necessary and could helpfully be achieved [26].

### 3.3. CNT and Graphene

#### 3.3.1. Definition and Technologic Trends

CNT corresponds to scrolled graphene sheets which are composed of juxtaposed hexagonal cyclic sp2 hybridization carbon atoms [13]. Since discovery in 1991 [3,137], carbon nanotubes (CNTs) have aroused a high amount of interest in their use as building blocks for many new applications based on outstanding mechanical properties [12] and which can be combined with their other interesting chemical and physical solid-state properties especially for various electrical, and opto-electronical devices and future integrated circuits due to their outstanding electrical, mechanical, thermal, opto-electric and solid-state combined surface properties [138,139].

Improved performances achieved with several of their specific properties, are well illustrated with the development of carbon-based material field emissions beginning with a-C:H, improving with doped diamond and ta-C and achieving much more performing results with CNT, which are used for atomic force, scanning tunneling (STM) and magnetic force microscopy [140,141,142,143,144,145]. They could improve performances of catalytic nanomotors [146] and many new applications could be developed after controlling synthesis conditions, size (diameter/length) and structure (chirality, semi-conducting/metallic properties, single or multi-walled) [147,148]. However, many difficulties appeared for industrial application owing to contamination, defect and various still often little understood effects yet [63,64] (such as rippling, and phase transformation) for which we propose some clarification next.

#### 3.3.2. Early Fundamentals on Graphene and CNT

Solid-state quantum mechanical calculations could predict many electronic level and phonon modes distribution for a well-defined carbon material bulk structure, which elementary optoelectronic properties and Raman frequency could be determined especially for diamond, graphite and graphenic materials [117,149,150,151]. However, more complex often not well understood Raman spectra have been experimentally observed for thin film carbon materials containing different phases (carbon composite materials), which have been mainly used as spectroscopic fingerprints of corresponding materials. Those could be first used for the empiric observation of their structure modification and for which refined aspects give more precise information [113,137,138,139].

**Figure 1:**
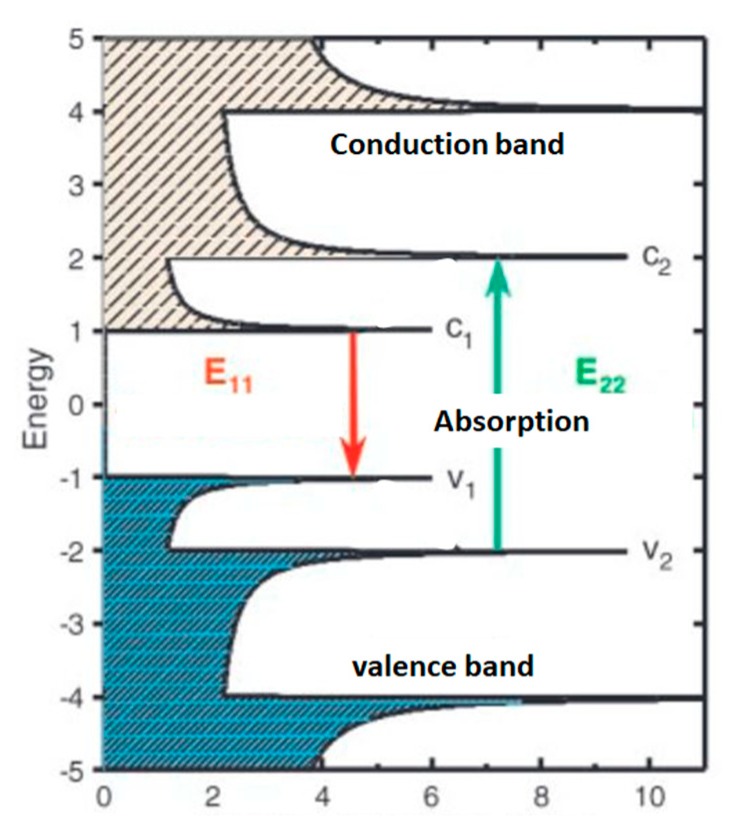
Scheme of optoelectronic band structure in single walled carbon nanotube by Bachilo et al. [158] reproduced with permission of the Journal of Science.

Considering some quantum electronic confinement effects for nano particles, very fine opto-electronic structure modifications are observed (Figure 1). This is especially the case for one and two-dimensional materials, which will be very sensitive to physical adsorbtion and chemisorption and to any bulk and surface structure modification [152,153,154,155,156,157,158].

They can be functionalized and will offer, for instance, selective IR optical response to molecular adsorbtion [159,160], which can be used, for instance, for selective photosensitive intrinsic polarimetry [161]. Additionally, improved electric conductivity can be obtained [162,163,164] with incorporation of iodine or copper, for instance, and also providing possible conduction anisotropy of expected interest for electric energy storage and antistatic encapsulating [165,166]. Very low resistivity is achieved with the electron ballistic transport effect, similar to superconductivity [167,168] being actually well understood in using revisited description of super-conductivity with electron/phonon synchronic gating during which no electron/electron and electron/phonon scattering occur [29].

All these results will be also very sensitive to radiation damage [169] material contamination and defect content [149,150,151,170,171,172] (whenever with questionable conclusions we will discuss in next chapter) and which in addition, are also depending on specific material structure edge configuration and possible thermally induced modification [153,154,155] (as discussed in more details in Section 5 and Section 6). However, interpretation and characterizing anomalies could be identified with the refined Raman theory [26], with which several observed contradicting aspects could be sorted out. Important new aspects of carbon phase transformation could especially be identified [25] with competing opposite effects that we believe can broadly explain the MEMS devices quality and performance distribution, which need to be comprehensively mastered and we briefly review next.

## 4. Brief Review of Quantum Activated Atomic Rearrangement

### 4.1. Atomic Rearrangement during Synthesis of Carbon Materials

#### 4.1.1. Graphitic Thermal Degradation and Diamond-Like Material Reforming

Graphite being the ground state of carbon, any carbon material can be degraded to more graphitic material by thermal effects ruled by the Arrhenius law and with low energy thermal activation. This effect is observed during longer thermal annealing of diamond, DLC, a-C:H and glassy carbon [3,15,27,111,112,113,114,115,116,117,129,130,131,173] below their sublimation temperature (~3500 °K) [174] (corresponding to ~0.3 eV electronic activation).

However, it is also known, that higher quantum electronic activation produced by different means and especially with photonic activation which can strongly influence chemical synthesis [175]. This is also the case during the formation of diamond and diamond-like material, which are not only formed with temperature/pressure and ionic thermal spikes producing compressive stress [111,112,124] but also with many other effects and without energetic ions (flame depositing, hot filament, high flux low ion energy, plasma jet, UV, X rays, electric neutralization energy, catalytic effects) [25,26,27,119,122,176,177,178,179,180] and especially the chemical recombination energy release (CRER) of C–C (~7 eV) H_2_ (~5 eV) and N_2_ (~12 eV) [25,121,181], which are exciting electrons with relatively high energies (>1 eV up to more than 10 eV compared to thermal activation in the ~0.1 eV range) before producing phonons and heat, which can produce a noticeable higher quantity of rearranged sp3 material structure. 

**Figure 2:**
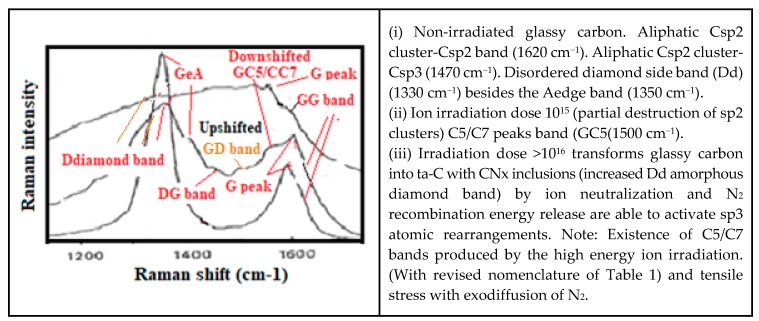
Glassy carbon 25 KeV N+ ion irradiated with additional annotations from M. Iwaki et al. [182] with permission of the journal of J. Mater. Res.

With Figure 2 we reproduce the Raman spectra from an N+ ion irradiated glassy carbon, which is transformed into a diamond like ta-C. To be observed that the annealing of CNx with a lower content of dissociated N will not produce the sp3 rearrangement effect because of insufficient N2 CRER activation events [182,183].

Updated Raman spectroscopy [26] shows that in many published thermal processing of carbon-based material supposed to produce graphitic material, in fact, resulting materials are also containing DLC, amorphous or nanocrystalline diamond [129,130,131,176,183,184] (what can be verified with measured sp2/sp3 obtained with electron energy loss spectroscopy and other more accurate, easy and fast XPS /Auger characterizing method, with which the measurement of the intensity of the Auger peaks of an XPS spectrum corresponding to Csp2 and Csp3 does not need any hazardous peak fitting assumptions and no deconvolution ) [27,120,121,122,123]. In such a case appearance of isolated diamond crystallites can correspond to increased abrasive friction. The diamond like phase transformation is also well documented, with a brief high temperature annealing of polymeric material that can be transformed into a diamond-like glassy carbon [132,133], meanwhile a longer annealing at higher temperature will produce a graphenic glassy carbon [133].

#### 4.1.2. Surface Polarization and Diamond-Like Atomic Rearrangement

It has been evidenced that adsorbtion energy can be directly transferred to the valence band electrons of the substrate, which after excitation are decaying with possible emission of corresponding photons of the same energy [185]. Thus, the adsorption of atoms and molecules on semiconducting material can produce electron/hole pairs and can produce a transient electric field (consequence of different mobility of electrons and holes being activated by released adsorbtion energy and which are then decaying [100]). In addition, it has been shown how an electric field transverse to a graphitic carbon thin film can enhance its sp3 content [186].

**Figure 3:**
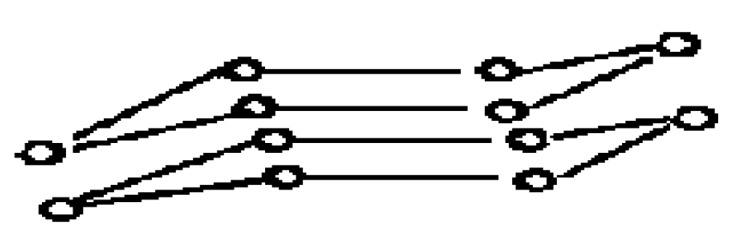
Transformation of graphene into H6 diamond with electric field polarization perpendicular to the graphene plane [25]. H6 diamond.: Stack of chair shape carbon hexagonal rings (sp3 bonded to four others). Raman frequency at~1325 cm^−1^ according to McNamara et al. [116] and Spear et al. [189] and Phelps et al. [190].

Above some electric polarization threshold energy, the carbon material can be transformed in a metastable material of higher density of cohesion energy. The hexagonal planar sp2 ring can be transformed in a buckled hexagonal chair plane (Figure 3) [25] (Similar to Silicene obtained with Si epitaxy on graphene [187], which is suggested to correspond to the caned structure of some carbon nanowires with irregular diameter [188].

Most carbon material types have more or less a semiconducting electronic band structure and metallic CNT are in fact semimetal materials with valence band and conduction band [27,134,149,150,151,191]. Therefore, atomic rearrangement in consequence of more energetic electronic activation and formation of internal electric field with different decay time of activated electrons and holes (higher energy quantum electronic activation) can appear in many solid-state systems.

#### 4.1.3. Criterion of Quantum Electronic Sp3 Activation 

**Figure 4:**
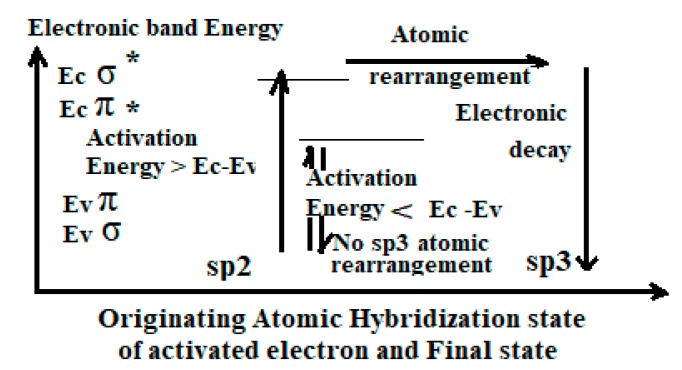
Principle of quantum electronic activated atomic rearrangement by S. Neuville [25].

During an atomic rearrangement (Figure 4), the activated electron must always occupy authorized energy levels. Achievement of ordered atomic rearrangement can only happen with fulfilled conditions [25,26]:

(1) Valence band electrons must be excited up to the conduction band of the initial and final state.
(2) More electrons must be activated than atoms to be rearranged.
(3) Atomic rearrangement can only be achieved with its local (proximity) activated electrons.
(4) The kinetic and density of activation events must be compatible with the decay and diffusion kinetic.

#### 4.1.4. Ordered Atomic Rearrangement and Stress Modification

Stress reduction is a major concern, in so far that internal stress can strongly affect adhesion and bulk diffusion [27]. Longer thermal annealing at a higher temperature produces graphitic degradation and stress relaxation, meanwhile a shorter thermal treatment of DLC, can reduce the stress without degrading the material as long as the limit of thermal stability has not been overpassed and the activation energy is not higher than the phase transition barrier potential [25,27,111,133]. This thermal stability depends on the structure of the carbon material (~900 °C) for polycrystalline diamond [117], ~600 °C/700 °C for hard ta-C [121,122,123,124,125] and less than 200 °C during the ta-C growth, considering that the flux of energetic ions bombarding the growing film surface during an arc deposition generates additional heating [111] and local transient higher temperature than on the substrate holder.

At a higher temperature than the thermal stability limit, the material will be degraded into more graphitic material, unless a higher electronic activation is able to counterbalance the graphitization process and which can then enhance the diamond-like character of the considered material. For instance, during hard UV irradiation [122,176] and with chemical recombination energy release (CRER) of H in a growing diamond film [25,192,193] and during glassy carbon synthesis, when the thermal process time did not exceed the time at which exo-diffusion of all hydrogen content has been achieved and the material has not yet be fully degraded to a more graphitic material [132,133].

With the exodiffusion of recombined H_2_ (or other gaseous material), tensile stress can appear [27,130]. However, uniformly distributed sp3 atomic rearrangement is generally forming higher packing density and stress relaxation, when no other effects such as thermal dilatation and/or ion peening is hampering this stress reduction process. This is typically achieved with catalytic sp3 rearrangement [194,195]. Catalytic effects produced with boron atoms can reduce C–H binding energy and for which consequently the H_2_ and C–C recombination energy release will be higher [25]. This explains why the added Boron in a high temperature diamond deposition process (where thermal dilatation can compensate the reduction of packing density) can produce better diamond quality in addition to the doping effect providing better electric conductivity [3,114,125,195].

#### 4.1.5. Examples of Diamond Like Atomic Rearrangement of Graphenic Material

Several other illustrative examples can be cited for the diamond-like atomic rearrangement:

##### CNx Annealing with Formation of Diamond Crystallites

During the annealing of some CNx materials where larger amounts of N_2_ CRER can exist, many nano-diamond crystallites appear [181]. This is only achieved when many dissociated nitrogen atoms exist, otherwise only few N_2_ recombination events will be produced and at a higher annealing temperature the material will be degraded into some more graphitic material [183,184].

##### Graphene Transformation into a Dielectric Material 

Adsorbed hydrogen on graphene has been reported to convert highly electric conducting graphene into a dielectric buckled 2D planar graphane [196,197]. However, the existence of graphane is questioned with different arguments. The so-called No-Go criteria of the Landau phase transition theory [198] predicts that graphane cannot exist for thermodynamic reasons. This effect appears to be very important to be accurately sorted out for many CNT/graphene NEMS applications.

On a graphene surface, C–H bonds are expected to be formed only on external graphene flake edges or on internal edges of voids and vacancies where dangling bonds exist, considering that the C–H bond energies (between 4 and 4.5 eV) is lower than the H_2_ binding energy (~5 eV) and much lower than the graphene or diamond C–C bond energy (~7 eV). Whenever the existence and stability of graphane was predicted by theoretic calculation [199], the question will be how this material can be formed, considering the low adsorption energy of the H_2_ molecule on a graphene surface and the low chemical reactivity of graphite and graphene for many chemical compounds (such as Cu etc.).

Considering the low adsorbtion energy on a defect free graphene surface, H_2_ molecules have a high surface mobility, except on the graphene defects (vacancies, voids, Smith Wales defects –C5/C7 odd sp2 ring pairs [200] where adsorbed H_2_ can be transformed in local C–H). Considering that H can easily recombine to H_2_ (~5 eV), the formation of alternating the C–H bond (~4.3 eV) on each graphene flake face will be unlikely. However, with the high H_2_ recombination energy release graphene can be transformed into a buckled H6 diamond (according to Figure 3) with enough available activation events and in agreement with observed formation of Csp3 [197] and looking in more details to the corresponding Raman spectra, with a Raman peak H6 structure at ~1325 cm^−1^ [199,200]. It can be observed that high pressure H_2_ on a graphene surface, can hardly be dissociated except on defects where dangling bonds exist, explaining why only local diamond structure can be expected [201].

##### Transformation of Graphene into H6 Diamond with N and O

Another demonstrative experiment suggests that graphane cannot exist. This is given when a similar transformation of graphene into a dielectric material with other atoms than hydrogen, and also, which is observed with nitrogen and oxygen. Quantum electronic activation can be produced with the N_2_ recombination energy release, similarly to what can be produced during some CNx annealing as precedingly reported [181] with the formation of diamond crystallites. The phase transformation can be enhanced in comparison to a similar experiment with H, because the N≡N recombination is releasing higher energy (~12 eV) than the H_2_ recombination and because the C–N bond energy (~3.16 eV) is weaker than C–H (~4 eV up to 4.5 eV) [25]. Binding energy differences of N_2_, C–C and C–N also explain in contrast to the early quantum mechanical calculated prediction [202] why the c-C_3_N_4_ formation is unlikely (because the C–C and N_2_ bond energy are much higher than C–N) and why this virtual material cannot be harder than diamond considering the equivalence of hardness with the density of cohesion energy [27].

However, graphenic G-C_3_N_4_ can be formed with the substitution N during a graphitization process and with the assembling of CN radicals. A material which is thought to be suitable for photo-catalytic H_2_ production from water [203]. However, we suggest that similar conditions can be expected with the same optoelectronic gap with more stable material corresponding to doped ta-C-like material, and also, which can combine the appropriate gap, electric conductivity and enough thermal/chemical stability.

##### Oxygen Sensitivity of CNT Electric Conductivity

Strong sensitivity to the oxygen environment of CNT appears to be a major problem to be solved for many NEMS applications [204]. Anticipating on the next Section 4 and Section 5 and in making usage of the revisited Raman spectroscopy, very demonstrative experiments with oxygen plasma etching of multilayer graphene are showing how on nearly defect free graphene sheets (no Raman C5/C7 band and no D peaks of any kinds) an important part of the graphene multilayer is transformed into an H6 diamond [205] and optoelectronic and electric conducting properties will be significantly modified. For such case the CNT cylindrical geometry is preserved because diamond structure islands are only formed on vacancies and around void edges.

### 4.2. Importance of Diamond-Like Phase Transformation on Carbon Material Properties

#### 4.2.1. Phase Transformation and Internal Stress Formation

Rapid and strong phase transformation toward atomic denser packed material can induce tensile stress before forming cracks, all the more this effect can be enhanced with the exodiffusion of lose bonded gaseous material (H_2_ or N_2_ or CH_4_ as observed for instance with the synthesis of porous glassy carbon). This is also observed during the annealing of some H-rich a-C:H or N-rich CNx materials [130,131,177] when some denser packed material can be formed containing diamond crystallites and for which higher friction can appear.

This sort of mechanism is suggested to explain how energetic UV will destroy graphene and CNT when these materials are subject to phase transformation toward isotropic denser packed sp3 with formation of cracks [206]. An effect which is also concerning metallic alloys, which can become brittle with cracks formation when incorporated hydrogen recombines to H_2_ and this effect is the consequence of different structures with different atomic packing density (corresponding to different oxidation states), which are also used in aeronautical and nuclear metallurgy [207].

#### 4.2.2. Incidence on Carbon Film Nucleation

Vacuum film nucleation is generally produced with disordered randomly distributed condensation, surface migration and cluster formation before being coalescent. If strong interlayer bonds are formed on the substrate surface or on the growing material surface, then surface migration is hampered and the structure of this first layer will be generally amorphous with reduced atomic packing density, especially when the growing film material is subject to ion bombardment which is heating the growing material and which will keep atomic disorder [208] except if the epitaxial order can be imposed and/or if the material can be activated towards better crystallinity. The first grown atomic layers is generally graphitic either amorphous or recrystallized in the form of Csp2 clusters depending on the achieved surface mobility of the condensing atoms on the substrate. This surface mobility can be very low in the case of strong bond formation corresponding either to diamond materials (~7.02 eV) or to graphenic materials (~7.03 eV).

However, nucleation of the diamond material appears where the chemical recombination energy release (CRER) activation is sufficiently available, which can be produced in larger quantities with different means: In the grooves of the substrate surface after scratching [25], which can be enhanced when instead of SiC bonds, higher energy C–C bonds can be formed and all the more, when clean contaminant free epitaxial conditions exist [111]. This is the case also, for heteroepitaxial conditions when additional activation energy can be delivered. For instance, with the diamond growth on an activated carbon pretreated crystalline molybdenum [119]. (This is no longer observed after decay of the surface activation) [111].

Graphene can be formed on a substrate with high surface mobility of condensed carbon atoms, which will have reduced adhesion on the substrate [13,172]. Meanwhile, single walled carbon nanotubes can nucleate on some catalytic substrate, with which some activation (much lower than necessary to produce diamond) will determine radius and chirality of the CNT [13,150].

Defects on the substrate, contamination and temperature can modify nucleation conditions and the adhesion of the growing material. The formation of interface bonds with higher binding energy on defect edges and causing electron excitation with released recombination energy, may produce some phase modification towards denser material structure, stress and additional defect formation [15,150,151,152,153]. Discontinuities, defects, vacancies and voids will be important to consider, when on their edges strong interface bonds can be formed, contrary to chemically neutral defect free graphenic surface.

#### 4.2.3. Incidence on Carbon-Based NEMS Engineering

All precedingly mentioned effects concerning phase transformation, adhesion, heat and electric conductivity, and also, which are depending on the type, size and number of defects are expected to cause major problems in industrial implementations of NEMS [10,13,14,15,21,22,23,24,63,64,147,148]. They will affect the life time and many opto-electronic and sensor functions, friction, wear, geometric tolerances, adsorbtion and mechanical properties.

Therefore, the principle of these effects must be well understood and correctly characterized. In all cited cases, it appears important to know and to master the types and number of defects and for which many interpretations will have to be revisited with all newly discovered effects, which need to be considered and to be correctly characterized, and what we discuss next.

## 5. Brief Review of Revisited Carbon Raman Spectroscopy

### 5.1. General Aspects of Carbon Raman Spectroscopy

**Figure 5:**
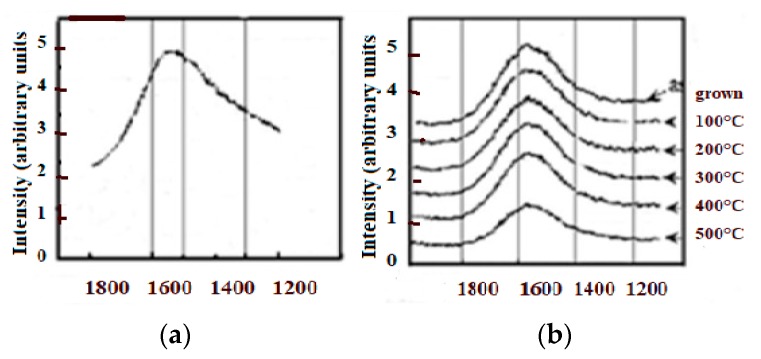
Raman spectra of stress shifted amorphous carbon graphitic carbon, ta-C and degraded ta-C (DLC) with no Csp2 clusters (no G sharp peak). Band max corresponding to (overlapping bands neighbor to the G peak at nominal 1580 cm^−1^). (**a**) Tensile stress ~30 cm^−1^ downshifted spectrum of amorphous graphite by Rouzaud et al. [208] by permission of the journal of Thin Solid Films. Additional dangling Csp2-Csp2 and C5/C7 bands. (**b**) Compressive stress ~80 cm^−1^ upshifted spectra of ta-C thermally degraded to less hard ta-C with reduction of stress. By Anders et al. [209] by permission of the Journal of Thin Solid Films.

The Raman spectroscopic assignment and interpretation still present in the literature of many confusing and contradicting aspects need to be sorted out. Carbon materials are often more composite than originally assumed and for which persisting grave lacks on Raman fundamentals must be cured. A distinction must be made between the D diamond that peak normally at ~1330 cm^−1^ and the so-called D-disorder peak which is normally at ~1350 cm^−1^ when no stress-shift applies. It must be determined which stress is to be considered and to which substructure the observed Raman band corresponds (Figure 5) [208,209].

**Figure 6:**
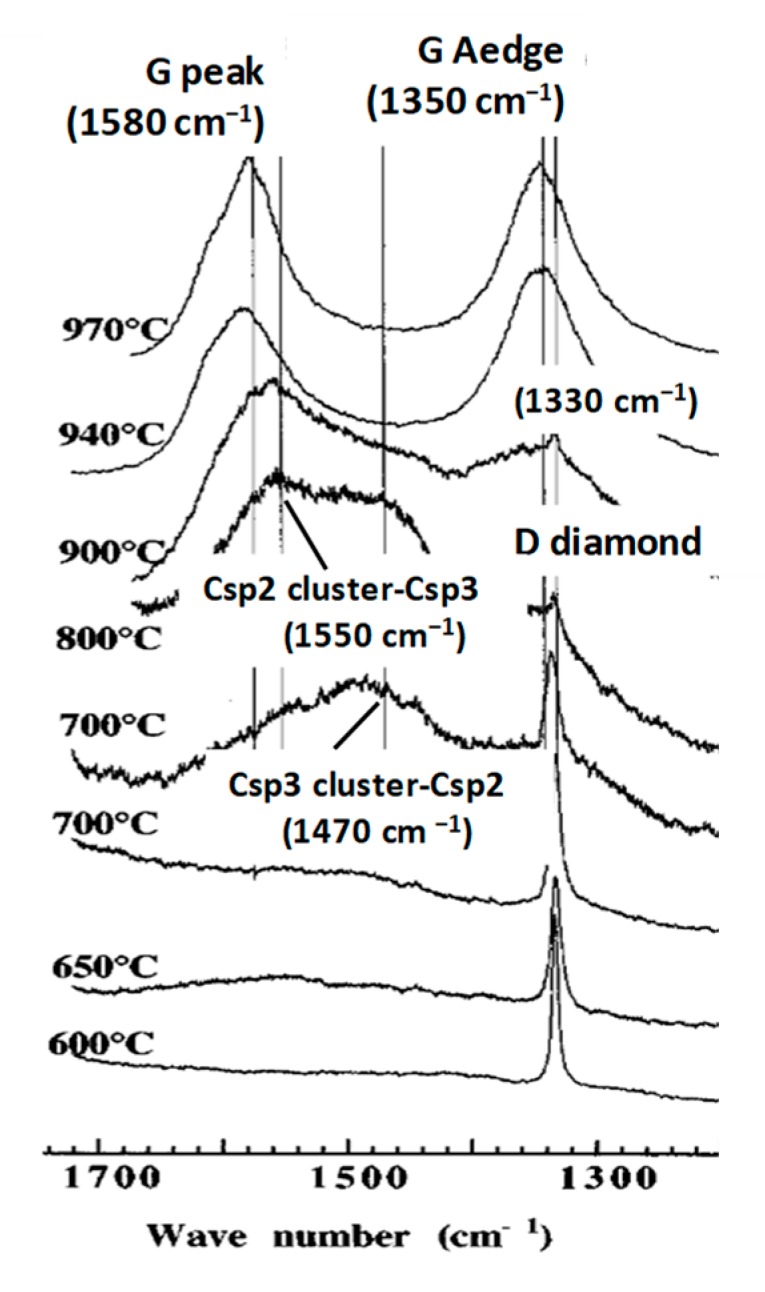
Diamond degradation with temperature by L. Fayette et al. [117] with permission of the Journal of Physical Review B. The D diamond peak decreases meanwhile Csp3-Csp2, G and G A edge peak are growing.

The analysis of the Raman spectra contrary to popular belief [210,211], cannot be limited to the sole measurement of the peak intensity and width of the respective so-called D and G bands [27,111,112,113,114,115,116,117,147,148,149,150,151,170,171,210,211], except when the material is either polycrystalline graphite [212] or diamond-like pure [113,173] (containing only sp3). This is well shown with Raman spectra, which have been recorded during a diamond annealing process, with which different peaks and bands appear, corresponding each of them to some specific substructure (Figure 6) [117]. Contrary to the elder persisting popular belief the sp2/sp3 ratio and the diamondlike character of carbon will generally not be able to be deducted from the ID/IG ratio [27].

### 5.2. Carbon Structures to Be Considered with Raman Spectroscopy

#### 5.2.1. Comparison of Raman Spectra from Different Carbon Materials

Different Raman peaks corresponding to different carbon material structures have been calculated with theory [189,190] and in agreement with experimental results from: (a) Micro-crystalline and amorphous diamond [116,173], (b) degraded variants and diamond-like carbon containing different kinds of subdomains [111,115,117,125,181]: Amorphous sp2/sp3 mixture, and amorphous/nanocrystalline sp3 parts) [130,173,209,210] (d) deformed sp2 clusters, graphene planes and graphenic fibers and tubes [134,150,151], (e) glassy carbon (porous mix of fullerenic GLC, graphenic material, diamond-like material and H6 hexagonal diamond) [111,112,113,132,133] and for which persisting confusion in literature needs to be sorted out.

**Figure 7:**
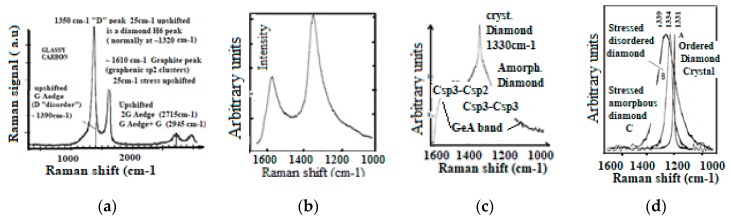
Comparison of Raman spectra of carbon materials that are supposed to be very different. (**a**) Glassy carbon (25 cm^−1^ stress up-shift) by Badzian et al. [132] with permission of Les Editions de la Physique. Glassy Carbon (GC) is not always graphitic. In the present case, ordered Diamond H6- (sharp up-shifted peak at ~1350 cm^−1^). Upshifted G peak (~1610 cm^−1^). Upshifted Ddisorder (GeA) (1370 cm^−1^) and G + GeA (2945 cm^−1^). Note similitude with b. (**b**) Diamond crystals imbedded in graphenic matrix by McNamara et al. [116] with permission of the Journal of Diamond and Related Materials. G peak, D diamond broad peak superimposed on GeA band (so-called D disorder band), (**c**) Superimposed D diamond peak with D diamond band and GeA band by Mc Namara et al. [116] showing existence of DG band (~1450 cm^−1^) and DD band (~1140 cm^−1^) with permission of the Journal of Diamond and Related Materials.. (Necessity of differentiating the D designation. (**d**) Comparison of (A) ordered diamond (B) disordered diamond (C) amorphous diamond broader peak (B) and (C) with stress shift by P.H. Huong et al. [173] with permission of the Journal of Diamond and Related Materials.

The Raman spectra of carbon materials that are supposed to be different, can show a paradoxical similitude for instance between the ta-C and amorphous graphite (Figure 5) or between the micro-diamond [115] and glassy carbon [132,133] (as shown in Figure 7). However, the stress shift must be considered in order to correctly assign the considered peak (or band). In Figure 6, the band maximum will not correspond to a shifted G band (which is normally at ~1580 cm^−1^), but to a band which normally would be observed at ~1500 cm^−1^ and which corresponds to a Csp3cluster-Csp2 band [25].

The D band designation had been extrapolated from the Raman peak of the single crystal diamond with a similar frequency (whenever different) and which contrary to elder belief is not always giving account for the sp3 concentration [26]. Proportionality to the sp3 concentration is only given when the carbon material is corresponding to totally mono- or poly-crystalline and to amorphous diamond materials [113,124,126,173]. For GLC (graphite like carbon) IG/ID has been shown (by Tuinstra and Koenig) to be proportional to the graphite crystallite size and surface/volume ratio [213] and which can be corroborated by the optoelectronic gap dependence on number of adjacent hexagonal cyclic sp2 rings [214].

#### 5.2.2. Raman Stress Shift and Atomic Disorder Band Broadening

It was generally admitted that a Raman D peak in graphenic materials containing no sp3 sites would correspond to the atomic disorder [112,170,208,209,210] and an assignment which can be questioned in taking into account following considerations

The disordered material has distributed an interatomic distance explaining the Raman peak broadening [26], considering interatomic energy potential U = αx^6^ and binding force Bf = 2αx^4^ and internal stress (which affects the interatomic energy potential) [215,216] it can be deducted:

δω/ω_0_ = η. δx/x_0_ (η a proportionality factor, ω the Raman frequency and δx the interatomic distortion to the ordered material interatomic distance x_0_ and with δω/ω_0_ = 6(1 − ν)/E_0_ (ν Poisson coefficient, E_0_ the mean elastic and 6 the stress). Therefore:

(a) Sharp Raman peak corresponds always to an ordered structure.
(b) Atomic disorder corresponds to peak broadening
(c) Raman shift is proportional to internal stress.

This suggests incoherent and confusing designation between the so-called D diamond and so-called “Ddisorder” band which appears necessary to be sorted out. It must first be taken into account that the stress shift of Raman frequencies is of greatest importance to be considered in order to avoid possible confusion between neighbor carbon Raman peaks.

A Raman peak/band might be erroneously assigned when the relevant local stress shift has been ignored. Additionally, awareness of possible atomic rearrangement may help to sort them out.

**Figure 8:**
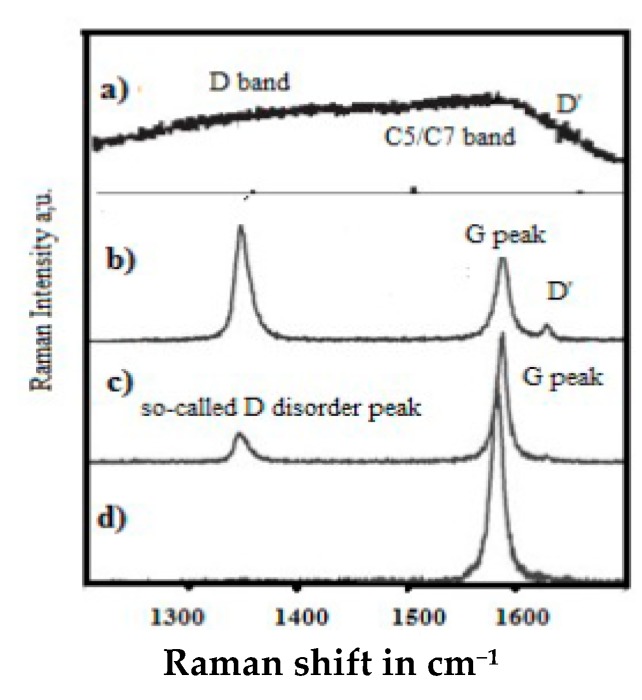
Relation between graphene void defects produced by Ar ion bombardment and so-called D disorder carbon Raman peak. By M.S. Dresselhaus et al. [170] with permission of Phil. Trans. R. Soc. A. (**a**) With Ion dose >10^15^ destruction of hexagonal ring. Disorder band broadening, with ~30 cm^−1^ stress shift. The broad “G” band corresponds to the upshifted band of C5/C7 odd rings. (**b**) D’ peak suggested to be assigned to dangling Csp2-Csp2 formed on internal void edges. (**c**) For ion dose 10^11^ the G peak is stress upshifted, without disorder band broadening, suggesting few incorporated vacancies. (**d**) Pristine graphene is an ordered structure (one sharp G peak).

The relation between some so-called D and D’ disorder peaks and atomic disorder in a graphene plane has been proposed with a study on defect formation in a graphene plane [170] (Figure 8).

However, such a disorder is only appearing for larger ion doses for which the graphene material is amorphized and hexagonal cyclic rings specific to graphene will be almost destroyed, meanwhile is to be emphasized that the appearing sharp so-called “D disorder” peaks correspond to well defined frequencies and which can only be associated to well-ordered material structure. Therefore, suggesting that this “D disorder” peak is associated to void formation with Ar ion impact, which has perforated the graphene sheet and void internal edges have been formed.

#### 5.2.3. Raman Peak Designation and Atomic Disorder

With the temperature annealing of some polycrystalline diamond film showing a single intense D diamond peak, a polycrystalline graphite structure appears with several peaks corresponding to specific subdomain structures for intermediate states of the graphitic degrading process [117].

Sp2 clusters and graphite crystallites are growing in number and size, meanwhile the diamond polycrystalline structure is reduced.

Csp3-Csp3 and Csp3-Csp2 dangling bonds appear on diamond crystallite edges and boundaries corresponding to the transition to graphitic clusters and graphite crystallites. Thus, also, Csp2-Csp3 and Csp2-Csp2 dangling bonds will appear on graphitic particle edges and boundaries (Figure 6) [25,117,210]. The D peak is growing, although the material is becoming graphitic and the sp3 content is decreasing, and the higher content of sp2 and graphenic particles have appeared. Obviously, this growing “D” peak is different from the D diamond peak and used to be called the “D disorder” peak, although no disorder peak broadening is to be considered.

**Figure 9:**
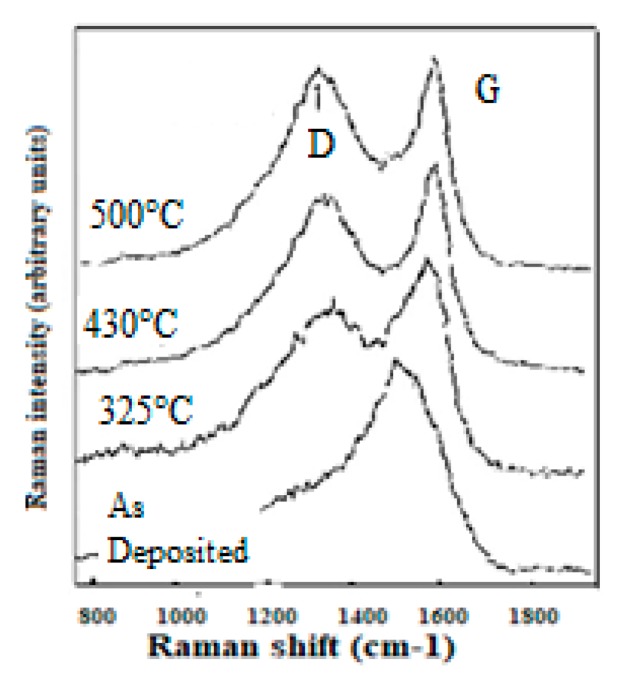
Annealing of H rich a-C:H. As grown, a-C:H ~70 cm^−1^ tensile down shift owing to H_2_ exodiffusion. With annealing, stress is reduced and Csp2 clusters grow (G peak) A GeA band (Csp2 cluster edge) appears beside a growing diamond peak (~1325 cm^−1^) (an H6 diamond). By Wagner et al. [130] with permission of Les Editions de la Physique.

With thermal annealing of some particular CNx and a-C:H [130,181] (Figure 9) for which graphite recrystallization would have been expected, it can be observed as a similar spectra than for the polycrystalline diamond [115,116] (Figure 7) (with a G peak for sp2 clusters inclusions) and similar, as well to some glassy carbon [132]. Here, a D peak/band (~1325 cm^−1^) is superimposed on a D disordered/amorphous diamond band and on a broader “Ddisorder” side-band, before being converted into graphite (carbon ground state) [113,130,211]. With a 500 °C longer annealing (1 h range) any stress is reduced and all observed Raman peak frequencies correspond to their nominal assignment.

### 5.3. Revision Necessity of Common Raman Scattering Description

Raman spectroscopy is widely used for graphenic material characterizing [217]. No so-called “D disorder” band is observed for the bulk of large graphite particles [117,212], in contrast to graphite dust [212] suggesting that the so-called “D disorder” peak corresponds to some edge effect.

**Figure 10:**
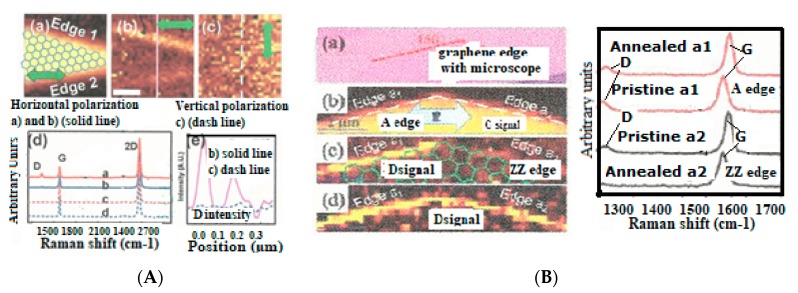
Micro-Raman showing locality on graphene A edges of the so-called D disorder Raman band. (**A**) Micro-Raman of graphene by Y.M. You et al. [154] with permission of Applied Physics Letters, Image of: (**a**) G peak, (**b**) “D disorder” peak (GeA peak) only on the A edge (**c**) GeA peak with vertical laser polarization. No D’ peak and C5/C7 band (no disorder). (**d**) Raman spectra on bulk. (**e**) Raman intensity geometric distribution of D signal (**B**) Micro Raman spectra showing graphene ZZ edge transformation into the Aedge with thermal annealing at ~300 °C adapted from Y.N. Xu [155] with permission of the ACS Nano. No C5/C7 band at ~1500/1530 cm^−1^. Pristine non-annealed graphene shows tensile downshift of ~20 cm^−1^ indicating that it was prepared by CVD. Stress disappears with annealing temperature at ~300 °C. (**a**) graphene edge location, (**b**) Image of G peak on bulk, (**c**) Image of D peak only on A edge after annealing, (**d**) Image of D peak on pristine graphene flake.

**Figure 11:**
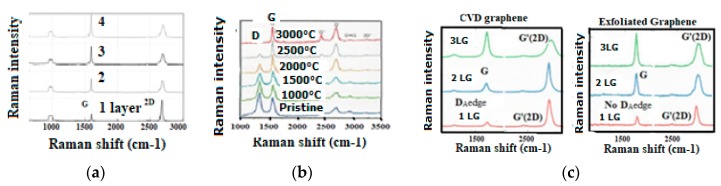
Raman spectroscopy of different quality of graphene. (**a**) High temperature longer time annealed graphene by Watanabe et al. [218] with permission of Diamond and Related Materials. No GeA peak (so-called “D disorder”). (**b**) Annealed graphene by J. Campos Delgado [172] with permission of Chemical Physics Letters suggesting many defects left, even after annealing. (**c**) Raman spectra by A. Reina et al. [219] with permission of Nano letters, showing no D disorder peak (GeA), sharp 2D and G on 1 L/2 L indicating reduced disorder for the exfoliated graphene and some disorder for the CVD graphene with broader G and G’ (2D) sheet without C5/C7 ring formation.

This is clearly evidenced with the high-resolution micro-Raman [154,155] (Figure 10) showing that the phonon vibration mode of the so-called Raman “D disorder” peak (~1350 cm^−1^) has a specific locality on the A edge, meanwhile this Raman peak does not appear on the ZZ edges. For some kinds of defect free bulk graphene, no D peak exist [170,218,219] meanwhile an intense 2D peak can be observed (Figure 11), depending on how the graphene was elaborated (many different elaboration processes exist today) [13,220,221]. 

**Figure 12:**
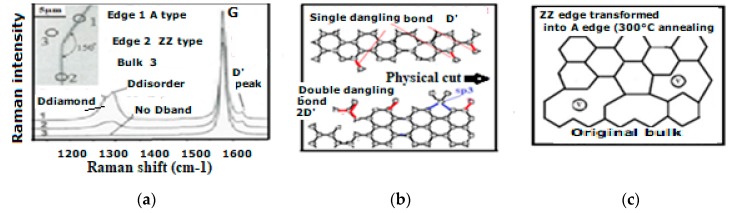
Graphene edge analysis of cut graphene plane and scheme of edge modification. (**a**) Raman spectra of cut graphene by Cançado et al. [222] by permission of the Physics Review Letters. D (1333 cm^−1^), D disorder peaks: GeA (~1350 cm^−1^) and D’ disorder Csp2-Csp2 (~1620 cm^−1^) are shown. Sharp G peak means that no disorder exists on hexagonal sp2 rings. (**b**) Our proposed scheme of graphene cut edge with the formation of sp3, single and double dangling Csp2-Csp2 corresponding to D diam, D’ and 2D’ peaks. (**c**) Our proposed scheme of voids formation associated to the thermally induced ZZ edge transformation into the A edge with internal ZZ and A edges on formed voids.

The Raman analysis and scheme of cut graphene (Figure 12) [222] can bring clarification on the corresponding structure of the so-called D’disorder band, considering that the Raman frequency for Csp2-Csp2 dangling bonds is the same than that for their IR stretching mode at ~1620 cm^−1^ and that such dangling bonds are likely existing after a graphene plane cut and noticing that the cutting process can involve compressive stress in the resulting edge structure [223]. 

However, in order to know more accurately the carbon structure of the material it is necessary to know to which structure the D peak corresponds and to consider the different types of subsystems including Csp3-Csp2 (~1520 cm^−1^ and ~1470 cm^−1^) [26,117,182], Diamond edge Csp3-Csp3 bonds ( often designated T band) ~1100 cm^−1^, H6 sp3 hexagonal Diamond ~1325 cm^−1^, D crystalline diamond/D amorphous diamond band ~1330 cm^−1^ [26,113,173]. Edge dangling bonds Csp2-Csp2 (so-called D’ peak) ~1620 cm^−1^) [170,222,223] (same IR and Raman frequency), C5/C7 odd rings ~1520/1550 cm^−1^ [224] (Table 1). For this achievement some revised aspects of the carbon Raman spectroscopy fundamentals are suggested to bring clarification and we recall next.

### 5.4. Refined Carbon Raman Spectroscopy

**Figure 13:**
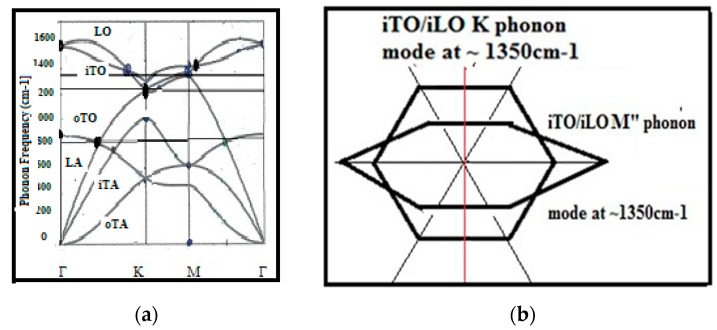
Phonon dispersion curves in graphene by Lazzari et al. [227] with permission of Physical Review B. (**a**) At 1350 cm^−1^ (the frequency of so-called Raman D “disorder” peak) two coupled phonon vibration modes corresponding to K and M modes and only the K mode is Raman active. (**b**) Representation of K and M coupled vibration mode for a hexagonal sp2 ring with perpendicular wave vectors. K mode corresponds to the vibration of A edge meanwhile the M mode is the vibration mode of the ZZ edge. iTO in-plane transverse optical mode, oTO out of plane transverse optical mode, LO Longitudinal optical mode, LA longitudinal acoustical mode, iTA in-plane acoustical mode, oTO out of plane acoustical mode

The classical Raman theory [225] has been refined with quantum mechanical aspects involving phonon modes distribution (Figure 13) and photon/electron/phonon scattering represented in the reciprocal space [150,151,226,227]. The Raman frequency corresponding to the homogeneous and ordered bulk structure could be satisfactorily calculated for diamond [189,190] and graphene [150,151]. However, this is not the case for the so-called “Ddisorder” peak as shown precedingly. It must be emphasized that among the different conditions which must be fulfilled, a Raman effect can only exist when the momentum and energy conservation laws are fulfilled [228]. The so-called “Ddisorder” peak was assigned to phonon backscattering on defects with the double resonance Raman effect. It is represented in the reciprocal space with the so-called “extra-valley” transitions between the Brilloin zones [150,151,170,226].

However, the usual quantum mechanical Raman theory [150] presents some flaws [229] in agreement with the point that the quantum mechanical theory generally neglects the locality of energy states and that the law of energy conservation is not always respected [26,98]. We state that the description of the double resonance back scattering on defects and edges and which is supposed to give account for the so-called “Ddisorder” peak (at ~1350 cm^−1^) does not fulfill the energy conservation law [26] (only the momentum conservation). Further on, the usual quantum mechanical description of the Raman effect is not considering the locality of the involved phonons. Therefore, the interference of the phonon being backscattered on a defect with the incident one (resulting into a stationary vibration modes) and the eventual coupling between phonon modes have not been considered either.

Considering that a wave vector in the reciprocal space corresponds to a wave propagation direction in the real space, we could refine this graphene Raman model with interferences of involved phonon modes and in taking into account the coupling between activated electrons and corresponding holes (the so-called Kohn effect) [230] and their different decay times in correspondence to each sort of scattering time [26]. The modified double resonance Raman effect corresponding to the backscattered phonon on edges and defects (we call “Coupled Double Resonance” CDR) can only exist when the backscattered phonon has the same direction with the original incident one (0° angle backscattering). Otherwise, the law of impulse conservation cannot be fulfilled. To be noted, that the erroneously so-called “D disorder” Raman peak (~1350 cm^−1^) in graphenic structures was also assigned for the breathing mode of a hexagonal sp2 ring [112]. However, this mode will be evidently quenched with higher number of adjacent rings [25].

Considering that this CDR Raman peak (~1350 cm^−1^) corresponds to an A-edge vibration mode as shown with micro-Raman [154,155] (Figure 10) and only to be considered on graphite hexagonal cyclic ring edges we have proposed the GeA designation for it (Table 1). It is here to be emphasized that the K mode vibration mode (of the A edges) is coupled to the M vibration mode (of ZZ edges).

**Figure 14:**
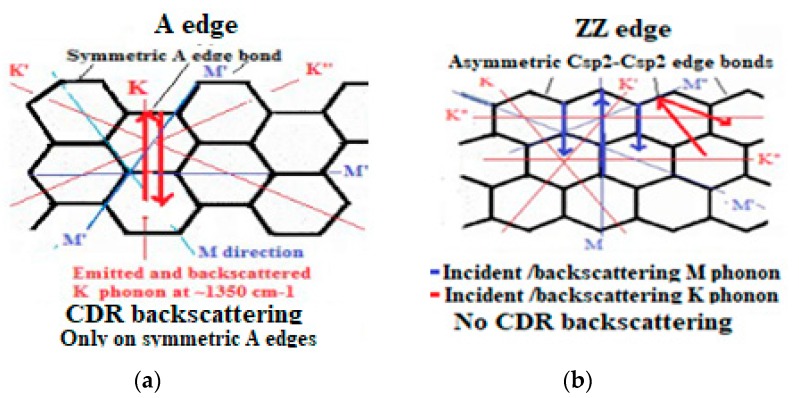
Phonon backscattering and Raman conditions at 1350 cm^−1^. (**a**) CDR Raman scattering conditions are fulfilled on a symmetric A-edge and for which in-plane K phonon at ~1350 cm^−1^ is perpendicular to A-edge. No CDR Raman scattering on asymmetric graphene A edge of respective K’, K” orientation. (**b**) No 0° angle backscattering on the ZZ edge for K and M phonon at ~1350 cm^−1^ where edge Csp2-Csp2 bonds are not symmetric to the K orientation. M phonon can be split in other phonon of lower energy and therefore, no CDR Raman at 1350 cm^−1^ can exist on ZZ edge.

The above described CDR model explains why the corresponding Raman effect is only appearing on the symmetric A edge on which a 0° angle backscattering is possible (Figure 14). 

This statement appears to be in agreement with the point that no equivalent Raman peak is observed either with non-symmetric A edges of the h-BN structure for instance [26,231]. This model will also explain why this so-called D “disorder” peak is only appearing on the smaller dust polycrystalline graphite particles and not for the defect free polycrystalline graphite bulk. This is suggested to be explained with signal/noise sensitivity and enhanced surface/volume distribution of relevant edge and bulk signals [26,212]. With this revised approach, different kinds of defect in graphenic materials can be characterized more accurately.

## 6. Defect Characterizing with Raman Spectroscopy

### 6.1. Phonon K Mode and M Mode Wave Scattering

From the phonon dispersion curves of graphene [150,151,226,227] (Figure 13) (corresponding also to some extent to sp2 clusters in DLC and to CNT, whenever related larger number of dispersion curves exists here [150], and considering coupled graphene sheet in-plane and out-of-plane vibration modes, it can be deducted that the G peak of graphite and graphenic material is only corresponding to the stationary vibrating mode specific to hexagonal cyclic rings (Γ mode) compatible with the vibration modes of adjacent material structures [26].

In the amorphous graphite (GAC) [208,228], no hexagonal cyclic ring clusters have been formed. Therefore, its broad so-called G band of the GAC material [208,228] cannot include the G peak corresponding to sp2 hexagonal cyclic ring clusters. It is the result of superimposed neighbor Raman peaks being broadened to a band by the atomic disorder.

This is likely the case for C5/C7 bands at ~1500 cm^−1^ (which is normally at ~1520/1530 cm^−1^) and for the adjacent band corresponding to Csp2-Csp2 dangling bonds of sp2 clusters at ~1590 cm^−1^ (which is normally at ~1620 cm^−1^) [26,223].

On such a Raman band, a stress shifted band corresponding to Csp2-Csp3 dangling bond of a Csp3 cluster is also observed at ~1430cm^−1^ (which is normally~1470 cm^−1^). A stress shifted band corresponding to a Csp3-Csp2 dangling bond of a Csp2cluster structure which would have been expected at ~1520 cm^−1^ (which is normally ~1550 cm^−1^) is unlike if no hexagonal cyclic ring can be considered, and when no G peak specific to them is observed.

For the so-called “Ddisorder” Raman peak frequency at ~1350 cm^−1^ two coupled vibration modes are identified on the phonon dispersion curves (Figure 13). The K mode (transverse to A-edge) and the M mode (diagonal direction in a hexagonal cyclic ring) (Figure 13b). However, according to theoretic predictions [150,151] only the K mode is subject to some Raman effect and can give account for the “Ddisorder” Raman signal with a double resonance backscattering, meanwhile the M mode will not (Figure 14). This explains why the so-called “Ddisorder” peak is not observed on the ZZ edges in agreement with the preceding discussion [154,155] (Figure 10).

An effect which is also confirmed with the Raman spectroscopy of the h-BN material for which no equivalent peak to the carbon D “disorder” peak exists [231], in contrast to the possible existence of an intense so-called 2D peak (2GeA) which is observed in some graphene Raman spectra which paradoxically are not showing any so-called D disorder peak (GeA) [218,219].

The addition of two M modes (complementary M’, M”) can form a new vibration mode with double frequency and the same direction than the K mode and for which a double resonance 0° angle backscattering can fulfill the impulse and energy conservation laws (thus, subject to a Raman effect).

The same is to be considered with the addition of two complementary K’ and K’’ modes, which can form a new vibration mode of double frequency with a wave vector perpendicular to an A edge and corresponding to a so-called 2D Raman peak (2GeA).

Therefore, the observed 2D peak can result from the addition of some vibration modes, in which each of them separately considered cannot produce a so-called D disorder Raman peak (A edge K mode). Thus, explaining the possible much higher intensity of the so-called 2D peak compared to the so-called “D disorder” peak [218,219] (Figure 11) and considering that the overtones of the so-called D disorder peak used to be weaker than the basic corresponding vibration mode.

### 6.2. Defect Types to Be Considered for NEMS Engineering

**Figure 15:**
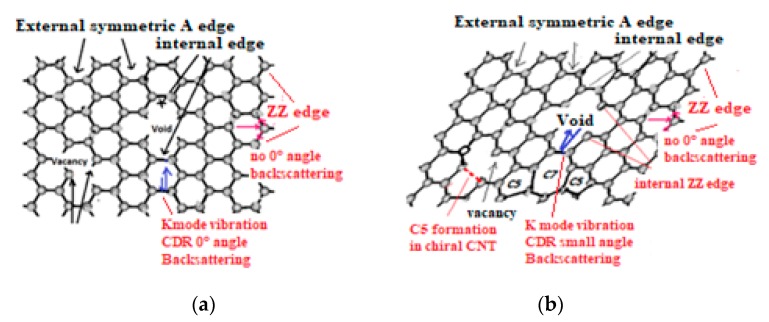
Defect in graphene bulk (vacancies and voids). (**a**) Phonon backscattering on external and internal A edges and ZZ edges in a metallic graphene. No Raman double resonance is possible on the internal edges of vacancies (ZZ edges) in contrast to voids where symmetric A edges exist. (**b**) Formation of C5/C7 odd rings and some internal A edges on vacancies of chiral graphene in consequence of chiral distortion. The reduced A edge asymmetry allows local Raman double resonance (in consequence of Heisenberg incertitude criteria).

Independent from those associated to doping (such as B and N) and contamination (such as H, O and N) and completing some description of defects in graphenic materials (multilayer stacking anomalies), several sorts of “intrinsic” defects have been identified which can appear in the form of network discontinuities in the graphenic bulk and on their external edges (Figure 12 and Figure 15) and which have significant consequences on NEMS properties [21,22,23,150,151,169].

- Vacancies and voids with different “internal” edges. Single vacancy has only ZZ edges and can be identified with “2D” (2GeA) Raman peaks when no “Ddisorder” (GeA) peak is observed or when the “2D” peak is more intense than the “D disorder” (GeA) peak [171] (Figure 11).
- Interstitials which can be rearranged during annealing in forming odd ring C5/C7 ring (SW defects) [151,200] with possible additional vacancy formation (Figure 10b and Figure 12c) [26].
- Edge discontinuities resulting for instance from graphene plane cut [222] can produce on the cutting edge single and double aliphatic Csp2-Csp2 edge dangling bonds at ~1620 cm^−1^ and 3240 cm^−1^) and which are corresponding to so-called D’disorder peak (Figure 12a). Those can evidently modify chemical reactions and material rearrangement on graphenic external edges.
- Graphene rippling has a focused high interest strongly affecting their mechanical and optoelectronic properties and their electric and heat conduction [232,233,234].

On a single vacancy of graphenic material, no internal A edge is formed and only internal ZZ edges exist. This suggests that the single vacancy will not produce any GeA Raman peak, contrary to larger voids which have both internal A and ZZ edges. In order to reduce the confusion between the D diamond and so-called “D disorder” peaks and very high impact on carbon-based MEMS and NEMS quality and performances (opto-electronical, electric and thermal conductivity properties and density of cohesion energy), we have suggested to modify the designation nomenclature with Table 1 [26].

Considering edge dangling electrons, easy chemical reactions with recombination energy release can be considered. Atomic and molecular species can be chemisorbed on vacancies (via their internal edges) and with which some new electronic band configuration can be obtained and which can determine some specific graphene functionalizing [10,11,21,22,23].

However, those can also produce sp3 atomic rearrangement in the surroundings of the vacancy/void if the released energy is able to produce sufficient electron activation. In such a case a dielectric material can be formed which will strongly affect the originally expected high electric conductivity.

### 6.3. Local Atomic H6 Diamond Rearrangement 

In addition to thermal transformation of the ZZ edge into more stable A edge [155] with the formation of additional vacancies (Figure 12c) [26], local diamond atomic rearrangement of graphenic materials can be produced with quantum electronic activation of higher energy, and with which graphene can be locally transformed into a dielectric H6 diamond structure [233] (Non shifted Raman peak at nominal ~1325 cm^−1^) corresponding to a buckled hexagonal structure containing only sp3 (Figure 3).

We suggest these being at the origin of the graphene rippling and which is much affecting the electric conductivity of graphene [234] and the mechanical properties of graphenic material. This is suggested to explain the higher stiffness of nano poly-crystal diamond, where the crystallite boundary material is containing graphenic materials which have been partially transformed in sp3 substructures and the whole diamond material is interlinked by more isotropic diamond material [235].

**Figure 16:**
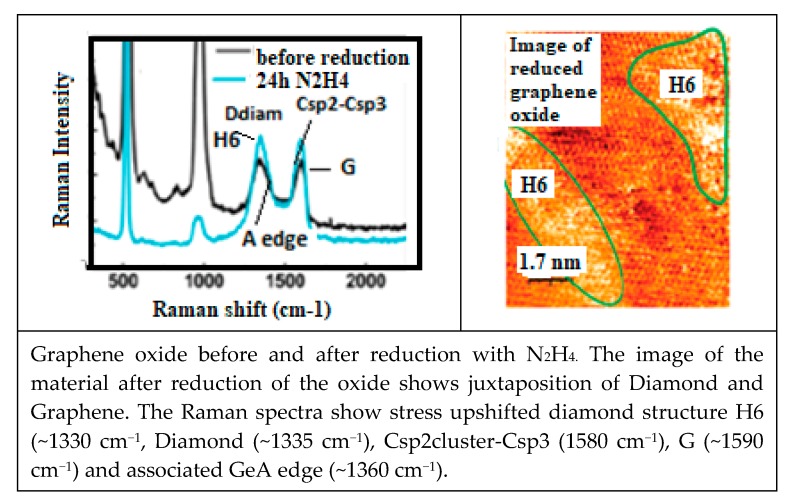
Graphene rippling and local H6 structure formation. Note: Reduced GeA edge after reduction and increase of H6 diamond by C. Gómez-Navarro et al. [236] with permission of the New Journal of Physics.

**Figure 17:**
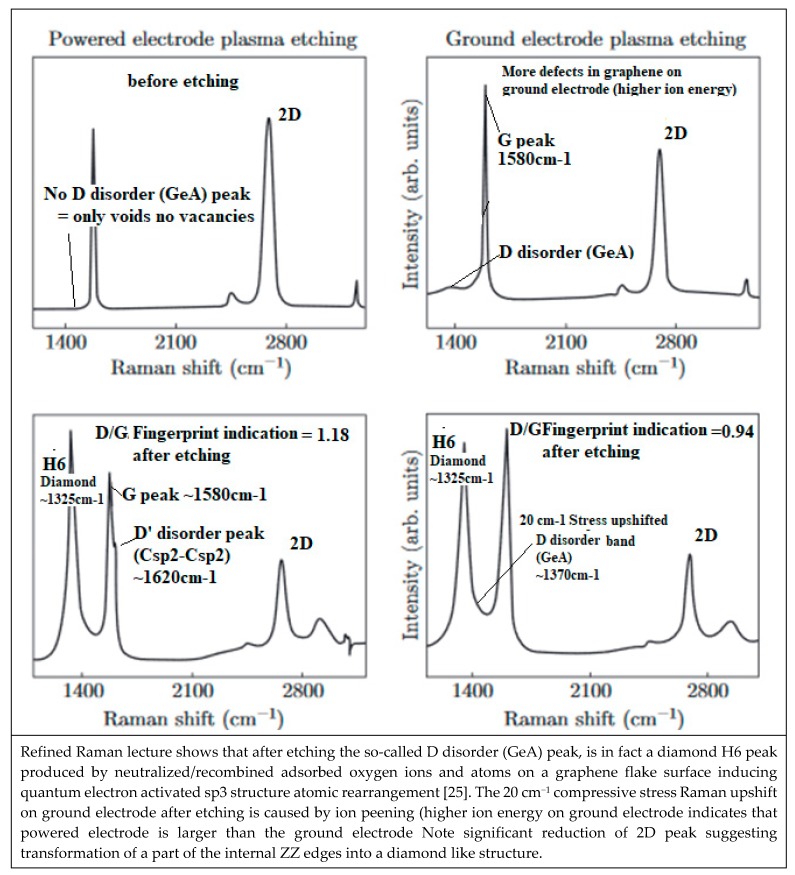
Raman spectroscopic results of oxygen plasma etched graphene by Al-Mumen et al. [205] with permission of Nano-Micro Lett.

**Figure 18:**
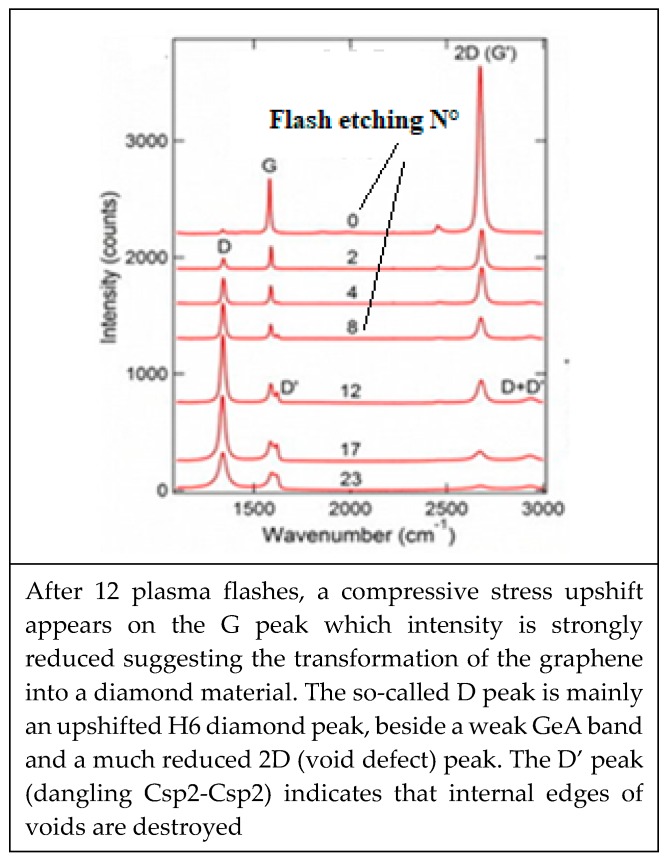
Example of revised interpretation of Raman spectra of oxygen plasma etched graphene by Childres et al. [237] with permission of the New Journal of Physics.

Refined lecture of produced Raman spectra is clearly showing that an H6 diamond structure has been formed (~1328 cm^−1^), meanwhile the G peak appears at ~1580 cm^−1^ (no longer compressive stress). Considering the reduction of graphene oxide, a process during which some O-X molecules are formed with corresponding higher energy release (X = H, O, etc.) [236] are able to produce some diamond like quantum electronic activation (Figure 16). This is also well shown with other experiments especially after oxygen etching of multilayer graphene [205] (Figure 17) and [237] (Figure 18) and with the modification of the sp2/sp3 content which can be evaluated from the plasmon spectra, and more easily with the sp2 and sp3 carbon AUGER peaks of XPS spectra [25].

In addition to defects which can affect the properties of graphenic material, the selective CNT adsorbtion efficiency (depending on adsorbtion energy) for molecular compounds of different shape must be considered, and which also depends on the CNT chirality and which are influencing the optoelectronic and mechanical properties and the volume and density of the formed CNT/adsorbate system. Recent high interest has been focused on the way to select the different types of SWCNT [238]. They can be separated with some selective buoyancy effects described elsewhere [239] and which appears of great importance for CNT based synthesis of complex helicoidal structures [11,81,82]. It is suggested to be at the origin of Precambrian abiotic synthesis of early NRA life molecules [240].

## 7. Application Developments

### 7.1. Role of Interface Structure on Composite Material Properties

#### 7.1.1. Anchoring and Adhesion of Graphenic Materials with Counter-Facing Materials

Graphenic materials have been first used for more performing load-bearing applications [64,65,241]. CNT powders are mixed with metal alloys, polymers or precursor resins to increase stiffness, strength and toughness and also heat and electric conducting properties. For instance, bulk graphene (reduced graphene oxide)-reinforced Al matrix composites with significantly improved mechanical properties in comparison to Al based alloys, and which is reducing cost, brittleness and cracks formation [242]. MWNT-polymer composites reach conductivities as high as 10,000 s m^−1^ at 10 wt % loading and is used for electrostatic assisted painting and which is thought to be used for anti-icing (with heat generated by electric resistivity) and microwave absorption [243,244].

However, these enhancements depend on nano particle size and shape, aspect ratio, alignment, dispersion and interfacial interaction. The last aspect will strongly depend on which type of chemical bonds can be formed on the internal edges of vacancies and void and on external edges of graphenic particles considering the discontinuities of the added graphenic material and on their number, size and density/unit area of vacancies and voids [200] and which need to be fully characterized.

#### 7.1.2. Modification of Intrinsic Mechanical, Electric and Optoelectronic Properties

Graphenic particle properties can be functionalized for specific and improved characteristics corresponding to more or less combined intrinsic mechanical, electric, optoelectrical bulk and surface adsorbtion properties and reactivity. This is to be achieved upon which kind of doping and which sorts of adjacent materials is in contact with the graphenic particle and which can affect the energy distribution of their optoelectronic band structure [101,102,144,158,162,195].

For instance, a graphene coating makes carbon aerogel composite particular elastic and resistant to fatigue [245]. Mechanical properties of high-aspect-ratio CNT can be tailored upon added silicon carbide coating [246], better performing transparent conducting electrodes can be achieved with transparent CNT sheet [243,244] and in using graphene silica composite [247]. Electric conductance could be strongly increased with added iodine and especially with copper doping [162,163,164]. Noteworthy the achievement of very high frequency nano transistors in making use of very low resistivity and ballistic electron transport condition in graphene sheets [167,168]. This, in analogy to superconductivity and which can be comprehensively described with a new model which consider the synchronic electron-phonon gating effect and the reduced fermi surface atomic rugosity and the reduced amplitude of transverse phonon in a graphene plane [29].

Transparent electric conducting mechanical and chemical resistant epoxy could be produced with magnetic molecule functionalized CNT [165]. More performing anode [166] and optimization of MWCNT/LIFePO4 cathodes have been achieved for Li-Ion battery [248]. Those are expected to be further improved with better compromise between electron conductivity and proton diffusion barrier properties [28]. 3D macroscopic carbon material scaffold with combined electro-mechanical properties could be developed [249]. Making use of combined piezo-electric properties, electro-mechanical resonator [249] and improved micro-loudspeaker could be produced [250,251]. Arrays of vertically aligned helicoidal CNT could be produced for better mechanical deformation for high frequency electric contact [252], Superelastic CNT aerogel muscles for bio-application [253].

However, these enhanced properties are strongly dependent on defect type, size and number and which must be characterized in order to be able to achieve some desired compromise between higher solid-state properties and precedingly described anchoring effect which is influencing the adhesion, the mechanical and chemical stability and other interfacial electronic effects. Notwithstanding, that eventual phase transition must be comprehensively kept under control.

#### 7.1.3. Local Activation of Phase Transitions on Edges and in Graphene Bulk

Incidence of high energy activation on phase transformation from graphenic material towards diamond material, could be many times unconsciously demonstrated for long. This can be stated in considering several corresponding Raman spectra features:

(a) With the differentiation between so-called “Ddisorder” peaks (GeA) and the neighbor collective vibration modes of the D diamond peaks and band (~1330 cm^−1^)
(b) The related Csp3cluster-Csp2 (~1470 cm^−1^) and Csp3cluster-Csp3 structure (~1150 cm^−1^).
(c) In considering some possible stress up- and down-shift which can be checked on the G peak.
(d) The differentiated analysis of the so-called G band which is not always containing a nominal G peak (corresponding to ordered graphitic and graphenic Csp2 material at ~1580 cm^−1^ when not stress shifted and which can be eventually broadened by disorder). They can correspond to the superimposition of neighbor shifted other peaks and bands [26,180,181,182,208,209].

Attention must be brought to the point that modification of mechanical properties of crumpled graphene sheet (graphene rippling) is not only the consequence of the new geometric non-flat surface shape [232,233], but almost basing on a phase transformation from a graphenic state toward dielectric H6 and diamond state (as we observe on corresponding Raman spectra). This phase transformation can be activated by various means (UV, external polarization, chemical recombination and ion and electron electric neutralization energy release) [25,234,235]. However, this eventual phase transformation will not be always homogeneously distributed, as observed for instance with the reduction of graphene oxide by Gomez et al. [236]. This explains the observed increased ohmic resistance (by mix of juxtaposed dielectric and highly conducting materials). In addition, some associated material shrinking is to be considered, which can cause cracks and increased brittleness and reduction of heat transfer capacity.

### 7.2. Friction and Wear

Some NEMS system includes rotating parts for which reduced wear rates and friction can achieve longer life time and higher number of mechanical cycles [254]. Mechanical robustness of a polymer substrate can be increased with an adherent graphene coating [255]. Considering the reduced chemical reactivity of defect free graphene surface, stronger adhesion of graphene on a polymer substrate (or reverse situation) is only possible with the formation of chemical stronger interfacing bonds on graphene vacancies/voids internal edge. However, on these spots, we suggest that released energy from chemical recombination of H_2_, C–C and new formed chemical bonds on the internal void edges can also transform the graphene material in the vicinity of these spots into a more wear resistant tribological diamond like material (which can be evidenced with corresponding Raman Ddiamond peak/band different from the so-called “Ddisorder” peak (GeA edge coupled double resonance vibration mode) [256].

These combined effects are suggested to explain why non-expected particularly strong reduction of wear and friction have been obtained on graphene coated metallic substrates sliding in dry nitrogen after the graphene coating begin to be corrugated with accumulation of sliding graphenic wear residues containing dissociated nitrogen [257]. This is to be considered with the very high N_2_ chemical recombination energy release, which can enhance the diamond like atomic rearrangement process [26]. To be observed that epitaxial graphene grown on SiC, can also be transformed into an epitaxial diamond structure. However, because of the much smaller interatomic distance in graphene than in SiC, interfacial atomic mesh mismatch and induced tensile stress is expected to form many more vacancies and voids. Thus, creating new additional stronger C–C interface edge bonds (~7 eV) which can enhance precedingly described effect [258].

### 7.3. Yarn and Scaffolds

High performing fibers and scaffold could be manufactured with the spinning of longer CNT and wires in considering pressure induced interlinking of adjacent CNT [259,260,261,262]. Enhanced fiber strength is obtained when fiber manufacturing is associated to polymer between the tubes which can better interlink the CNT via void defect anchoring effects [263,264] and also in causing diamond like atomic rearrangement in the vicinity of the voids (considering that simple vacancies will not be large enough for receiving all reactants necessary to such atomic rearrangement and with which stronger interlinking can be produced). However, such a process involving some phase transformation from graphene to diamond H6 phase may reduce its electric conductivity.

We suggest that the higher electric conductivity of Cu doped metallic CNT [265] is obtained when vacancies and voids can be filled with some atomic species able to form an electric conducting continuous flat CNT surface (similar to superconductivity conditions, as previously discussed).

For such application semiconducting CNT must be avoided, correct orientation of metallic CNT particles must be secured. Diamond-like atomic rearrangement forming more dielectric material must be avoided (or reduced to thin interlayer material, through which the electric conductivity is obtained by electron tunneling, all the more that diamond and ta-C materials have low work function). Addition of copper can fill larger voids with reduced chemical recombination energy release (Cu-C binding energy is very low) and with electron enrichment of the electron conduction band edges and in agreement with achieved high electric conducting CNT/Cu composite materials [163,164]. It will be essential here to optimize size, density and number of voids in the precursor graphene material and its contamination being reduced, considering that atomic rearrangement towards more dielectric materials has to be minimized. Using biochar material, a graphitic electric conducting carbon dust [266], its 3D isotropic distributed orientation is contributing to easier reproducibility of carbon-based composited materials [267].

### 7.4. Micro and Nano Interconnecting and Thermal Management

With the possibility of increased strength, elasticity, heat and electric conductivity and reduced copper electromigration, low weight and low cost, carbon-based fibers appear to be interesting materials for micro and nano interconnection and thermal management of many electromechanical and electronic devices [268,269,270,271,272,273,274]. With bottom up technology vertical tubes and fibers can be catalytical nucleated with strong bonds on its specific substrate alloy materials [275]. However, great care must be brought to the achievable low ohmic, strong and stable electric contact to other added parts of the NEMS. Any possibility to have the contact wire/fiber material being transformed into a low graded electric conducting material must be avoided. It must be controlled and managed (a) the diamond atomic rearrangement forming dielectric material by higher chemical recombination energy release (CRER) and (b) the surface passivation with reactive contaminants which can affect both the adhesion and the mechanical strength of the link and the resistivity of the electric contact. Cleanliness and selection of fiber type on defect content, composition, mechanical and electric properties will be a major concern.

### 7.5. Electronical and Optoelectronic Functions and Field Emission Effects

Different nano electronic devices combine [134] chemically and electric field modifiable semiconducting properties, low work function and different mechanical properties, which can be associated to high thermal conductivity, high current density and very high mobility, and ballistic properties, low voltage field emission properties and fast switching.

This could be achieved and/or improved with associated efficient chips cooling being up to ten times better than with copper and with which electromigration of copper can be avoided [274], especially in making use of polymer/graphene and CNT composites nano thin films [243,244,245,246] and with which different devices could be elaborated. Among them, flexible electronic integrated circuits and transparent thin film could be produced on transparent polymer and glass substrates, thin film transistors (TFT) and Field effect transistors (FET) [166,167,168,276,277,278,279,280,281]. Those are thought to be used for low power/low energy consumption field emission display [140,141] with which organic light emitting diode (OLED) can be activated [142] or selective light emission can be produced on optoelectronic functionalized CNT field emitters [142,143]. Here, particular care has to be brought to reduced contamination and defect contents and to possible phase transitions especially induced by various chemical recombination energy release activation.

### 7.6. Solar Cells, Hydrogen and Energy Storage and Energy Conversion

#### 7.6.1. Solar Cells and Energy Storage

**Figure 19:**
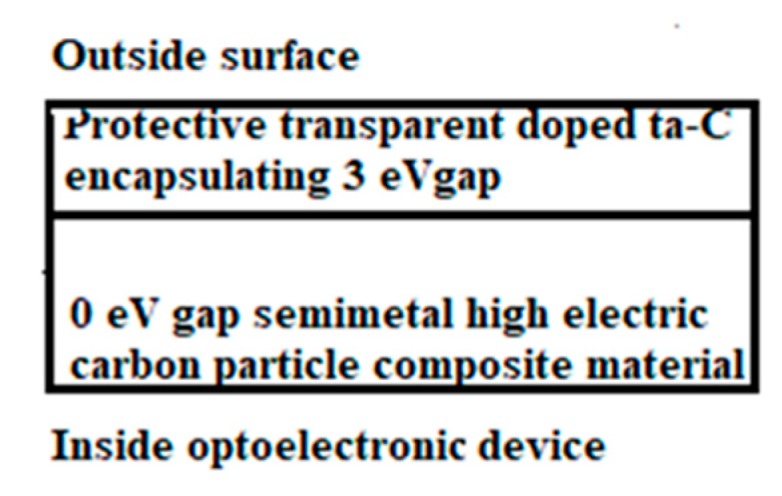
Scheme of conducting system with little conducting doped ta-C coating on highly electric conducting composite graphene.

Organic solar cells have focused much interest, since low cost flexible thin film photovoltaic system could be produced with them [282,283,284]. Graphene polymer composite could increase the conversion efficiency, in reducing electron/hole recombination, since electrons in the conduction band can be faster evacuated [285,286]. Such graphene polymer composite exhibit also transparent conductive properties which are thought to be used for displays and for solar cells, considering they are much cheaper than usual ITO (Indium Tin Oxide) and related TCO (Transparent Conductive Oxide) [243,244,247,282,283,284]. As a result of their extreme high electric conductivity, they can be used as transparent electrodes in very low film thickness. They can be deposited with spin deposition techniques without vacuum technologies. However, polymers are not best diffusion barrier and give little protection against humidity and low weight elements, and that thicker coatings can absorb a significant part of the solar light. Therefore, it will be useful to protect them with less permeating transparent ta-C encapsulating which can provide additional anti scratch resistance and resistance against hard UV [121] (Figure 19). Harder stress annealed transparent ta-C exhibiting very high antireflection in addition to other interesting properties, can generally enhance over the double of yearly solar light harvesting per unit surface, in collecting solar light with oblique incidence and especially azimuthal light during many days along the year where the sky presents some luminous coverage [287].

Association of different carbon-based materials have been considered for improved electric energy storage and energy conversion (fuel cells) [28,288,289,290]. Several major aspects suggested to be considered are the extreme differences in work function (from lower than 1 eV up to over 5 eV) between different categories of carbon materials and other properties which must be correctly distinguished. On a low work function graphene surface, H+ can be neutralized and recombined to H_2_ and induce phase transformation into more diamond like materials. Meanwhile, on a doped ta-C surface (highly diamond like) no chemo-structural change can be expected with such H_2_ recombination energy release (CRER) activation mechanism.

Thorough distinction between different types of carbon materials must be achieved, considering their different porosity and surface rugosity, chemical inertness, electric conductivity, work function and diffusion barrier properties (for instance homogeneous, harder, dense packed ta-C have best diffusion barrier properties including for H^+^ protons which other carbon materials will not have) [28]. Despite their poor electric conducting properties (whenever being doped), electric transport can be maintained through very thin ta-C layers by electron tunneling effect and can be used in multilayer nano materials with which low friction and high antiwear properties can be associated to sufficient electric conductivity for sliding contact brushes for instance [256].

#### 7.6.2. Hydrogen Storage and Photocatalytic Production from Water

Considering hydrogen in modern clean energy management, much interest has been developed for its storage efficiency and low-cost production [291]. Hydrogen can be stored at ambient temperature with much less energy than necessary for cooling and gas compression in making use of temperature dependent and reversible adsorbtion capability on CNT external and internal wall surface [292,293]. Physical adsorbtion of H_2_ on CNT is to be considered on its defects where dangling bond electron stay captive, meanwhile chemisorption corresponding to the formation of C–H bonds on the graphene surface and on its vacancies and voids internal edges (~4 eV) appears to be unlike, because the C–H bond energy is lower than the H–H bond (~5 eV) of a H_2_ molecule. Therefore, hydrogen storage on graphenic material will be much dependent from contaminants which can screen the defects [294]. With the dissociation of adsorbed H_2_ by catalytic and electrolytic effects [295], H atoms can be better chemically bonded to the graphene substrate defect edges. Consecutive temperature activated H_2_ recombination can release the stored atomic hydrogen in form of molecular H_2_. The desorbtion mechanisms is generally endothermic and is absorbing energy, meanwhile the H_2_ recombination and process is releasing energy with which the CNT material can be locally converted into some denser H6 diamond like material and will enhance the formation of defect with tensile stress. Process optimization will depend on defect characteristics.

Hydrogen production with photocatalytic dissociation of water has been thought in using the CNx material which can be tailored on its optimized optoelectronic gap (~1.8 eV) and with sufficient electric conductivity and with which solar light can dissociate water molecules with H_2_ formation [203,296]. Enhanced efficiency of similar process has been obtained with association of usual catalytic materials such as palladium/TiO_2_ material [295]. However, these materials can be degraded by oxidative processes which will harm to their operational life time. We suggest that chemically more stable doped ta-C which can also be tailored at 1.8 eV gap and presenting some anti-soiling properties might give the route for better satisfactory solutions.

### 7.7. Sensors, Medical Applications and Miscellaneous

Functionalizing of graphenic materials, assembled to yarn could produce electrosensitive elastic fibers thought to be used for artificial muscles [253] and highly performing bio sensors [68]. Several Carbon based NEMS sensor principles can be distinguished: Selective mechanical properties (cantilever and membrane deformation, mass selective resonance) [35,36,37,38,69,71], selective molecular adsorbtion of functionalized graphenic materials [297,298,299,300] and selective optoelectronic modification [158,159]. A model for biologic and artificial olfaction had been proposed based on the appearance of transient polarization pulse which can be produced by selective adsorbtion on specific molecular compounds wrapping an electric conductor [100]. With the functionalized graphenic material, its optoelectronic band organization is modified and can present IR fluorescence being activated by specific adsorbtion energy release, and an effect which is used for instance for the monitoring of glucose in the blood [159,160].

A reverse effect is to be considered, when some functionalized graphenic material is adsorbed on biologic cells and tissues which can have toxic and antibacterial effects and being used for cancer therapy [101,102,103,104,105,106,107]. CNT scaffold composed by strongly interconnected fibers [249,301] can be used for bone tissue engineering [302]. Noteworthy, is the possibility to produce some helicoidal molecular structure with the adsorbtion of proteins [303] with are expected to have specific sensor properties. The same effect is suggested to be at the origin of first terrestrial abiotic RNA synthesis [240]. Many other applications have been developed, using graphenic composite scaffold for water nano-filtering and purification [304], as flame retardant material which combines heat conducting cooling effect with formation of diffusion barrier material [305], plasmon enhanced detection of microwave [306], photodetection with broadband polarimetry, with functionalized CNT upon corresponding photon energies and making use of their electric conductance anisotropy [161].

For all these systems, it appears the decisive role of defects of the graphenic sensor materials on which functionalizing molecules will be anchored with modified optoelectronics. Contaminants can screen and modify the defects and the optoelectronic organization of the functionalized graphenic materials and quantum electronic activation by specific chemical recombination energy release can transform a graphenic/polymeric material into diamond and diamond like dielectric material. The last effect which is thought to be at the origin of manufacturing of high performing diamond/diamond-like composite material for abrasive resistant and more performing cutting tools with 3D printing of liquid polymer/diamond crystallite mixture [307].

## 8. Conclusions

Carbon-based materials have extreme material properties of interest for more advanced NEMS applications. However, despite huge progress particularly with them, their implementation has to face several hindrances [1,10,23,65]. Changes to electronic and mechanical attributes of carbon-based materials must fully be explored before their implementation, especially because of high surface area which can easily react with environments. The NEMS design must receive particular attention to contamination and actual material structure and defects which can affect adhesion, surface rugosity (rippling corresponding to phase transformation towards H6 diamond and other diamond-like structures), friction (especially on local asperities), electric conductivity, inter connection, optoelectronic properties and surface functionalization. It is necessary to be fully aware about them, and all aspects must be correctly characterized and selected upon their manufacturing processes and their origin in order to be able to optimize and secure achieved results.

For this purpose, revised fundamentals have appeared as essential. This is concerning on one hand the characterizing achieved especially with Raman spectroscopy and on other hand some dramatically neglected effects inducing phase transformation and significant modification of material properties. For instance, with phase transformations of graphene and polymeric material toward diamond and diamond like material by quantum electronic activation and which can be particularly efficiently produced by high energy H_2_, N_2_, O_2_ and C–C chemical recombination and electric neutralization energy release and which can produce diamond-like material glassy carbon and/or diamond particle inclusions and different kinds of amorphous diamond materials.

Diamond-like phase transformation appears to be very useful for many anti-wear and tribological application (effects being used for instance by Sandvik for the production of new diamond composite materials [307] or the contrary, when the main advantages of graphenic particles electric and opto-electronical properties have to be preserved and the appearance of defects and cracks by shrinking effects has to be avoided (similar effects exist also with different steel and aluminum based alloy when for instance with recombination of incorporated atomic hydrogen to H_2_ [207]). For this purpose, the efficient diffusion barrier properties against atomic hydrogen diffusion of very thin harder ta-C coating appear to be of high interest [121]. When being stress annealed without graphitic thermal degradation it is suggested to be used for different kinds of transparent antireflecting, anti-erosion, anticorrosion and anti-soiling encapsulation in association with transparent highly electric conductive polymers for many applications [287].

## Figures and Tables

**Table 1:** Raman nomenclature for carbon material characterizing.

Raman (cm^−1^) (Nominal)	Peak/Band	Type of Structure (Non-Stressed Structure)	Type of Bonds Energy in eV
~1330 ~1325 ~1200/1400	D peak DH6 peak Dd band	Ordered Diamond cubic Ordered hexagonal diamond Amorphous diamond, ta-C	Csp3-Csp3 ~7.02 ~7.015 eV Overlapping with DD band
~1150 ~1470	DD DG	Edges of Diamond crystallites. Diamond and sp2 edges	Aliphatic Csp3–Csp3 Aliphatic Csp3–Csp2
~1580 ~1560/1620	G peak G band	In plane double degenerated Γ phonon mode stationary vibration of sp2 cyclic ring Atomic disorder broadening	Csp2-Csp2 ~7.03 eV collective bond vibration Superposition GG, DG, GC5/C7
~1620 ~1510	GG GD	Csp2-Csp2 clusters edge in a-C and DLC Csp3-Csp2 cluster	Csp2-Csp2 ~7.03 eV collective bond vibration
~1490 ~1540	G_C5_ G_C7_	C5 ring C7 ring	Fullerene C5 (~1550 cm^−1^) (upshift by plane curvature)
~1350 ~1300/1400	GeA GeA band	A edge 0° CDR scattering Voids internal A edges 1st, 2d order disorder on edge Broadening by edge disorder	Free edges: not active with ┴ vertical polarized laser light Bonded edges: all laser light polarization
~2690	G_2P_	2 phonon CDR scattering Any polarized laser light	In plane 2K and 2M Preferential in-plane polarization
~150 ~1600 ~1560	RBM G+ G−	Breathing mode of CNT (radius dependent) CNT in plane Longitudinal CNT in plane Transversal	Collective phasic stretching Distorted Csp2-Csp2 by sp2 plane curvature of CNT
